# Heteroannulations of cyanoacetamide-based MCR scaffolds utilizing formamide

**DOI:** 10.3762/bjoc.21.13

**Published:** 2025-01-24

**Authors:** Marios Zingiridis, Danae Papachristodoulou, Despoina Menegaki, Konstantinos G Froudas, Constantinos G Neochoritis

**Affiliations:** 1 Department of Chemistry, University of Crete, Voutes, 71003 Heraklion, Greece

**Keywords:** 2-amino-substituted heterocycles, cyanoacetamide, Gewald reaction, multicomponent reaction (MCR), pyrimidine

## Abstract

C1 chemistry has a central role in the efficient utilization of single-carbon molecules, contributing significantly to sustainability, innovation and economic growth across various sectors. In this study, we present an efficient and rapid method for synthesizing a variety of heteroannulated pyrimidones using cyanoacetamide-based multicomponent reaction (MCR) chemistry. By utilizing specific MCR-based scaffolds as precursors and employing the abundant and inexpensive formamide as a C1 feedstock under neat conditions, we were able to efficiently access substituted thieno-, quinolino- and indolopyrimidones without the need of column chromatography. Further, a single-crystal X-ray structure was obtained, revealing certain geometrical features.

## Introduction

The term “net-zero carbon” is becoming increasingly common as we consider a future marked by a rising global temperature and severe weather patterns – a result of human-induced greenhouse gas emissions. The principle of net-zero revolves around the idea of using Earth’s carbon resources at a rate that does not exceed their natural replenishment. In 2015, the United Nations introduced the Sustainable Development Goals (SDGs), wherein the 7th goal focuses on ensuring access to affordable, renewable and clean energy [1]. Moreover, the European Union has committed to ambitious environmental targets as part of the European Green Deal [2].

Advancements in C1 chemistry are pivotal to achieving this equilibrium. As such, it is becoming increasingly essential for synthetic methods to align with a net-zero carbon future [2–3]. Therefore, advancing C1 chemistry remains a crucial endeavor for our group [4]. In synthetic organic chemistry, C1 compounds are usually installed using CO, CO_2_, HCO_2_H, CH_3_OH and CH_4_, which is mostly due to their presence in greenhouse emissions [5–6]. Although abundant and inexpensive, their valorization still remains problematic due to their thermodynamic stability and chemical inertness [7–15]. Multicomponent reaction (MCR) chemistry is a type of convergent chemistry characterized by diversity, complexity and efficiency. MCRs are compatible with C1 chemistry due to the generally great tolerance of different functional groups. They have been mostly employed in the synthesis of oxazolidinones and oxazinanones utilizing CO_2_ and CO [4,16–20]. In addition, carbonyldiimidazole-based heteroannulations via MCRs have been reported, giving access to drug like scaffolds [21–24]. However, their employment in C1 chemistry should and could be advanced.

**Figure 1:**
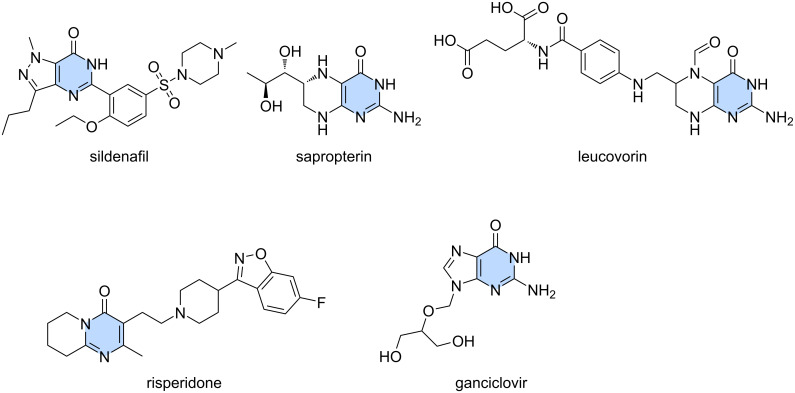
Heteroannulated pyrimidones in drug discovery: blockbuster drugs that are based on the privileged pyrimidine scaffold.

Herein, we point out formamide as an alternative relevant building block for C1 chemistry by using specific, suitably functionalized MCR scaffolds. This positions formamides as versatile, synthetic hubs towards privileged scaffolds and high-end chemicals. Over the years, the reactivity of formamide has been widely explored in heterocyclic chemistry, but it has only recently started to be established as a C1 feedstock. [25]. Its high polarity and dielectric constant (it is miscible with water) [26], with the ability to solubilize a wide range of reagents, from salts to polymers, proteins and saccharides, renders formamide an excellent C1 building block [25,27]. Thus, our target under the umbrella of C1 chemistry was to provide a straightforward access to the privileged scaffold of fused heteroannulated pyrimidones, which demonstrate a broad range of biological activities [28–35], including use as emissive nucleoside analogues [36–38]. Moreover, they are used in a variety of commercially available drugs (Figure 1).

## Results and Discussion

### Design and strategy

**Scheme 1:**
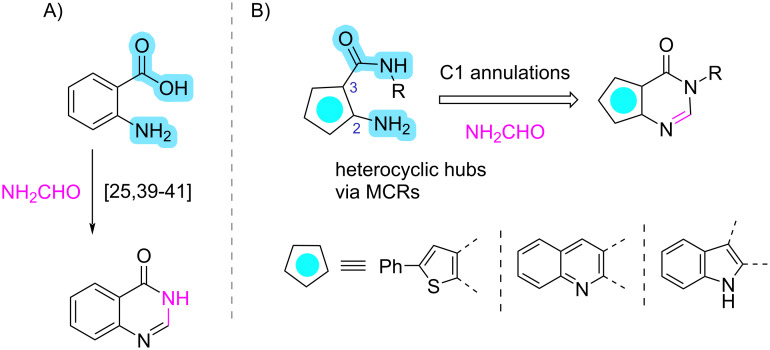
Strategies towards targeted adducts: A) Niementowski quinazoline synthesis utilizing anthranilic acid and B) access to heteroannulated pyrimidones by MCRs of suitably substituted heterocycles and formamide as C1 source.

We envisioned applying the Niementowski quinazoline synthesis [25,39–41] (Scheme 1A) by employing three different heterocyclic systems as precursors, which have both an orthogonally installed amino group and a disubstituted amide group at the 2- and 3-positions, respectively, and reacting them with formamide (Scheme 1B). Those synthetic hubs can be rapidly accessed by cyanoacetamide-based MCRs, which is an interesting reaction type that gives access to privileged cores and has been utilized numerous times in medicinal chemistry campaigns as hits, leads and eventually even drugs, such as 2-aminothiophenes, -quinolines and -indoles [42–45].

### Synthetic exploitation

**Scheme 2:**
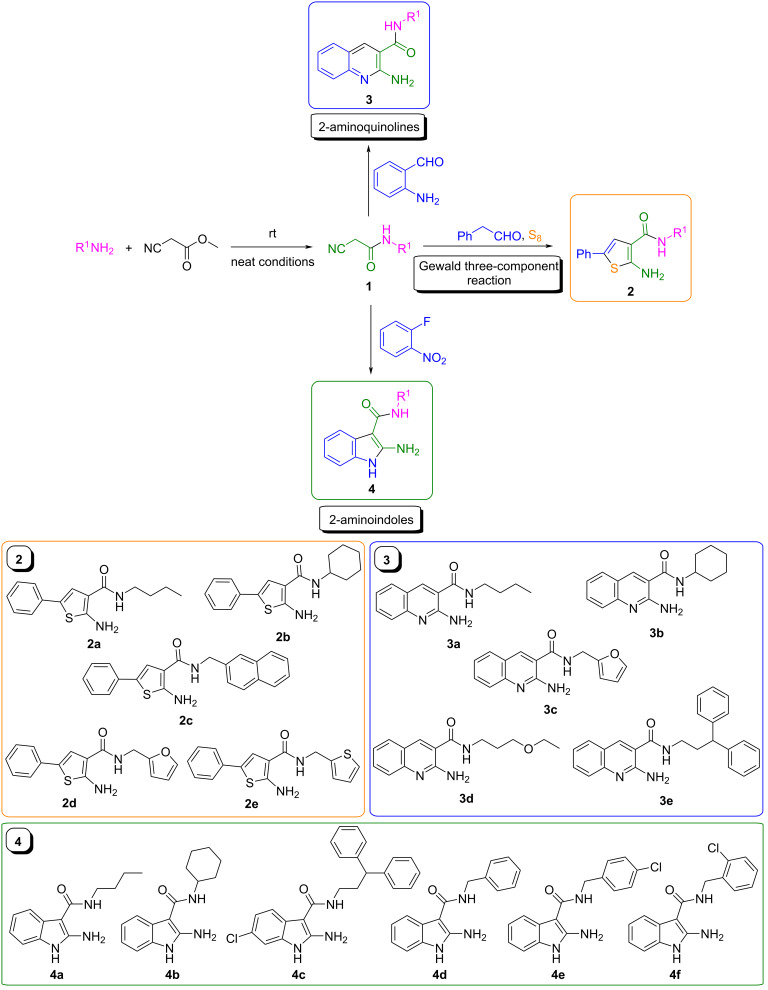
Access to the key building blocks **2**–**4** by employing three different nonisocyanide-based MCRs. Diversity and complexity were the essential features of our library of starting materials. The colored frames indicate the different scaffold types.

The synthesis of the key cyanoacetamide building blocks was our primary objective. In a parallel setup, a variety of primary amines was reacted with methyl cyanoacetate [46], giving rise to the corresponding cyanoacetamides **1** (Scheme 2). Subsequently, they were reacted accordingly to yield a variety of the targeted precursors, 2-aminothiophenes **2** [42], 2-aminoquinolines **3** [45] and 2-aminoindoles **4** [44], via Gewald three-component reactions. Our focus was to create a representative, diverse and structurally complex library of building blocks, covering a range of shapes and chemical spaces, to facilitate formamide-based heteroannulation for the synthesis of the desired adducts. Thus, we employed aliphatic and (hetero)aromatic, bulky and linear amines with different substitution patterns. The compounds **2**–**4** were purified by recrystallization and employed as such (Scheme 2).

To our great delight, the heterocycles **2**–**4** could successfully be subjected to refluxing formamide under neat conditions, instantly yielding the desired thienopyrimidones **5a**–**e**, quinolinopyrimidones **6a**–**e** and indolopyrimidones **7a**–**e**, respectively, as reported in the literature [42,44]. In accordance with the reported mechanism, after the initial formylation of the amino group at position 2, an intramolecular nucleophilic attack by the NH moiety of the amide group resulted in pyrimidone annulation [41]. This was observed for the first time and completed the reported heteroannulation landscape utilizing the Niementowski reaction [25]. In general, the reactions had a rather broad scope as a great range of cyanoacetamides was compatible. The synthesis was efficient and rapid as the final adducts could be isolated only by precipitation. In addition, the reactions were performed in a parallel setup using custom-made metal blocks.

**Scheme 3:**
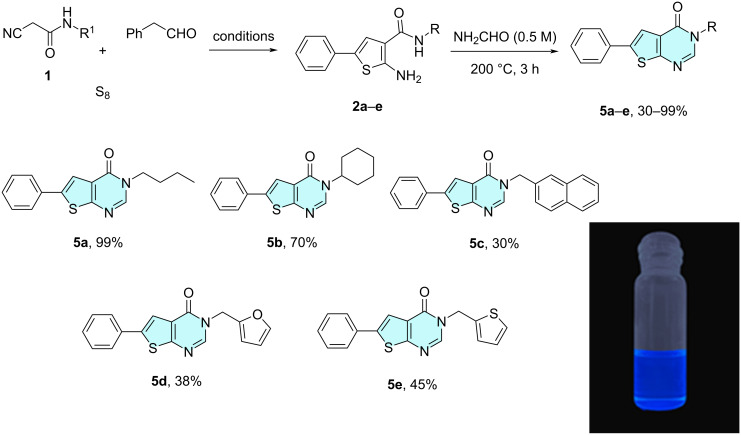
Synthesis of N-substituted thienopyrimidones **5a**–**e** by a Gewald three-component reaction employing 2-aminothiophenes **2a**–**e** and formamide as C1 source. A characteristic fluorescence for compound **5a** was reported (365 nm in DMSO). The reported yield refers to the overall two-step synthesis.

Specifically, thienopyrimidine and thienopyrimidone derivatives exhibit a range of biological activities, i.e., analgesia, anti-inflammation, antihypertension and many more (DB06889, DB07397, DB08777) [47–56]. Thienopyrimidone derivatives have also been used in the context of other Gewald-based MCRs in the past, but the reported diversity has been rather low [57–60]. The reaction of **2** with formamide was performed within only 3 h (Scheme 3), yielding the N-substituted thienopyrimidones **5a**–**e** in 30–99% total yield (2 steps), as well with aliphatic and (hetero)aromatic substituents.

**Scheme 4:**
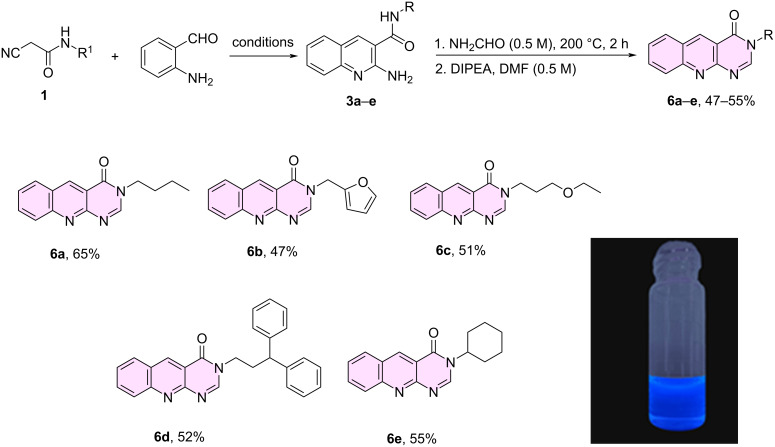
Synthesis of N-substituted quinolinopyrimidones **6a**–**e** from 2-aminoindoles **3a**–**e** and formamide as C1 source. A characteristic fluorescence for compound **6c** was reported (365 nm in DMSO). The reported yields refer to the overall two-step synthesis.

Quinoline derivatives are prevalent in nature, and many exhibit a range of biological activities, including antimalarial, antitumor, anthelmintic, antibacterial, antiasthmatic, and antiplatelet effects [61–63]. In particular, quinolinopyrimidine and pyrimidone derivatives have attracted a great interest due to their biological profile [64–69]. After some optimization, we determined that harsher reaction conditions than for the synthesis of **5** were required for the targeted adducts **6** to form. Additional treatment with DIPEA/DMF afforded the corresponding substituted quinolinopyrimidones **6a**–**e** in 47–65% total yield (2 steps) within 12–16 h. Notably, the products carried a series of aliphatic and aromatic substituents (Scheme 4).

**Scheme 5:**
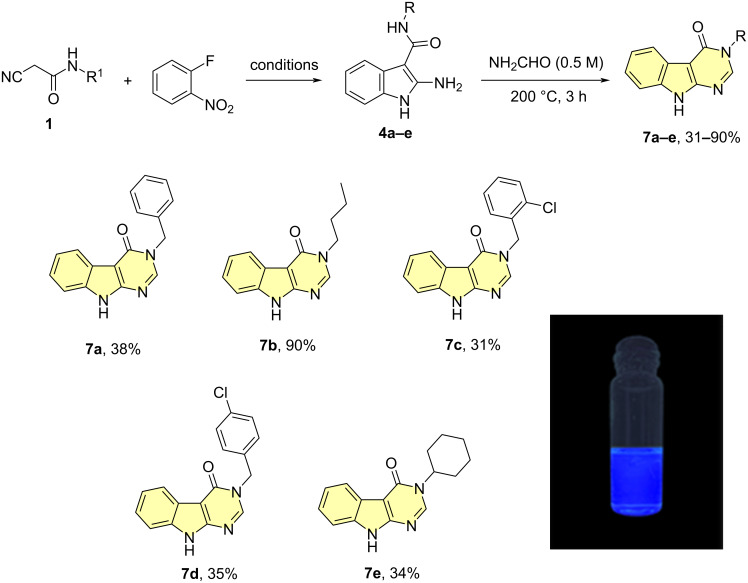
Synthesis of N-substituted indolopyrimidones **7a**–**e** from 2-aminoindoles **4a**–**e** and formamide as C1 source. A characteristic fluorescence for compound **7b** was reported (365 nm in DMSO). The reported yield refers to the overall two-step synthesis.

Pyrimidines and pyrimidone-bearing indole derivatives are crucial in organic chemistry because of their extensive use as bioactive compounds with a wide array of significant biological activities (DB03074, DB03304, DB08131) [70–72]. In a similar fashion, substituted indolopyrimidone derivatives **7a**–**e** were obtained within 3 h in 31–90% total yield (2 steps) upon heating with formamide (Scheme 5).

**Figure 2:**
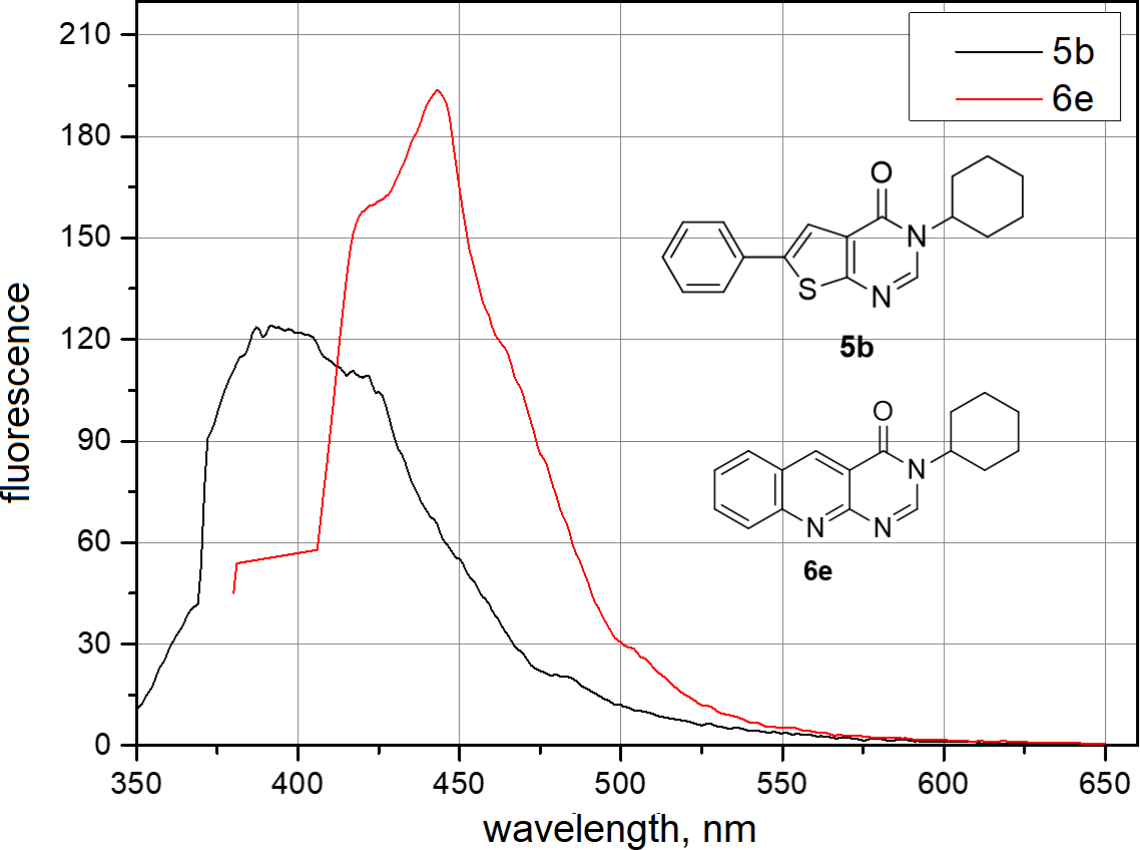
Representative fluorescence spectrum of compounds **5b** (λ_ex_ = 330 nm) and **6e** (λ_ex_ = 430 nm) at 0.2 M in DMSO.

Next, we characterized certain physicochemical properties of the newly synthesized compounds. The absorbance and emission spectra of **5**–**7** were obtained, showing similar profiles across all the different scaffolds (Figure 2 and Supporting Information File 1). The compounds **5**–**7** exhibited a maximum absorbance (λ_max_) at 330 nm, 284 nm and 310 nm, respectively, which also corresponded to their excitation wavelength (λ_ex_). Upon excitation, fluorescence emission was observed at an average wavelength of 384 nm for **5a**–**e**, 439 nm for **6a**–**e** and 430 nm for **7a**–**e** (see Supporting Information File 1 for detailed information).

**Figure 3:**
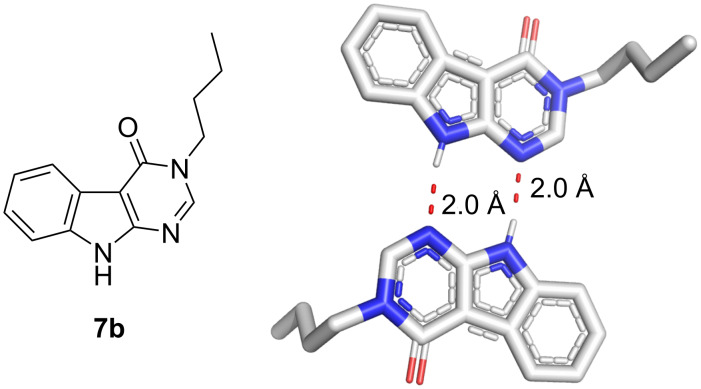
Molecular geometry observed within the crystal structure of compound **7b** (CCDC 2376493).

In support of the proposed scaffold **7b**, we were able to solve its crystal structure (Figure 3). An intermolecular bifurcated hydrogen bond network of 2.0 Å was revealed, demonstrating potential of these derivatives in drug and material discovery.

## Conclusion

In conclusion, we successfully combined the Niementowski quinazoline synthesis with nonisocyanide-based chemistry, thereby expanding and enhancing the scope of C1 MCR chemistry. That way, we obtained 15 diversely substituted heteroannulated pyrimidones, employing privileged thiophene, quinoline and indole scaffolds in a rapid fashion, without the need of column chromatography, in a parallel setup.

## Supporting Information

File 1 Experimental methods, synthetic procedures, analytical data and exemplary copies of NMR spectra of novel compounds.

File 2 CheckCIF report for **7b**.

File 3 CIF file for **7b**.

## Data Availability

Data generated and analyzed during this study is available from the corresponding author upon reasonable request.
