# Supplementary material for: Heteroannulations of cyanoacetamide-based MCR scaffolds utilizing formamide
Source: Beilstein J Org Chem. 2025 Jan 24;21:217–25. doi: 10.3762/bjoc.21.13 (PMC11773184; doi:10.3762/bjoc.21.13)
Supplement: File 1 — Experimental methods, synthetic procedures, analytical data and exemplary copies of NMR spectra of novel compounds. [file Beilstein_J_Org_Chem-21-217-s001.pdf]

BEILSTEIN JOURNAL OF ORGANIC CHEMISTRY

Supporting Information

for

Heteroannulations of cyanoacetamide-based MCR scaffolds utilizing formamide

Marios Zingiridis, Danae Papachristodoulou, Despoina Menegaki,
Konstantinos G. Froudas and Constantinos G. Neochoritis

Beilstein J. Org. Chem. **2025,** *21,* 217–225. doi:10.3762/bjoc.21.13

Experimental methods, synthetic procedures, analytical data and exemplary copies of NMR spectra of novel compounds

License and Terms: This is a supporting information file under the terms of the Creative Commons Attribution License (https://creativecommons.org/licenses/by/4.0). Please note that the reuse, redistribution and reproduction in particular requires that the author(s) and source are credited and that individual graphics may be subject to special legal provisions.
The license is subject to the *Beilstein Journal of Organic Chemistry* terms and conditions: (https://www.beilstein-journals.org/bjoc/terms)

Supporting Information

Table of Contents

1. Experimental methods and materials S2
2. Synthetic procedures and analytical data S3
General procedure for the synthesis of cyanoacetamides **1a**–**i**[1] S3
General procedures for the synthesis of the 2-aminothiophenes (GW-3CR) **2a**–**e**[2] and pyrimidones **5a**–**e** S4
General procedures for the synthesis of the 2-aminoquinolines **3a**–**e**[3] and pyrimidones **6a**–**e** S4
General procedures for the synthesis of the 2-aminoindoles **4a**–**e**[4] and pyrimidones **7a**–**e** S5
3. Exemplary copies of NMR spectra of novel compounds S12
4. Representative copies of ^{1}H NMR spectra of the MCR-based precursors S29
5. Procedure for absorption-fluorescence measurements S36
6. Single-crystal X-ray structure determination S37

1. Experimental methods and materials

All the reagents and solvents were purchased from Sigma-Aldrich, AK Scientific, Fluorochem, Abcr GmbH, Acros and were used without further purification. Thin-layer chromatography was performed on Millipore precoated silica gel plates (0.20 mm thick, particle size 25 μm). Nuclear magnetic resonance spectra were recorded on Bruker Avance 500 spectrometers {^{1}H NMR (500 MHz), ^{13}C NMR (125 MHz)}. Chemical shifts for ^{1}H NMR were reported as δ values and coupling constants were in hertz (Hz). The following abbreviations were used for spin multiplicity: s = singlet, d = doublet, t = triplet, q = quartet, quin = quintet, dd = double of doublets, dt = doublet of triplets, td = triplet of doublets, m = multiplet. Chemical shifts for ^{13}C NMR were reported in ppm relative to the solvent peak. High-resolution mass spectra were recorded using a LTQ-Orbitrap-XL (Thermo) at a resolution of 60000@m/z400. The Varioscan Plate reader spectrometer from Thermo Fisher Scientific was used to obtain the absorption and emission spectra.

2. Synthetic procedures and analytical data

*General procedure for the synthesis of cyanoacetamides **1a-i***[1]

Methyl cyanoacetate (15–30 mmol, 1.0 equiv) and the corresponding amine (15–30 mmol, 1.0 equiv) were added together into a 20 mL vial and stirred at rt. After 2 h, the precipitate is filtered and washed with cold diethyl ether, then dried on vacuum to give the desired products **1a**–**i** in quantitative yield (>95%).

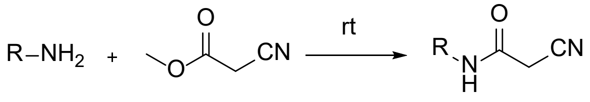

1a-i, >95%

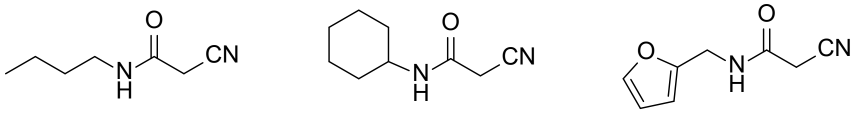

1a **1b** **1c**

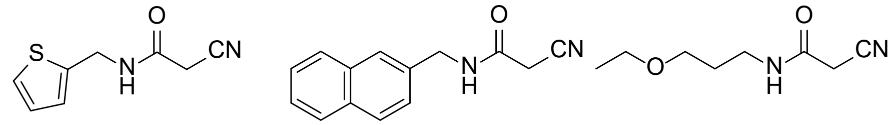

1d **1e** **1f**

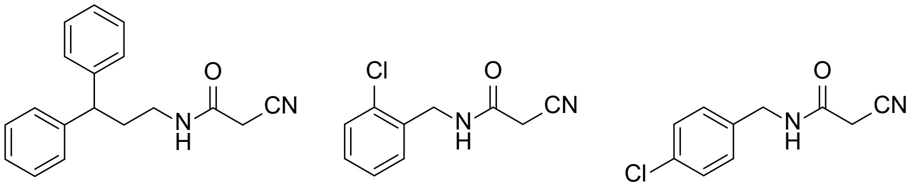

1g **1h** **1i**

General procedures for the synthesis of the 2-aminothiophenes (via Gewald three-component reaction, GW-3CR) ***2a*****–*****e***[2] *and pyrimidones* ***5a*****–*****e***

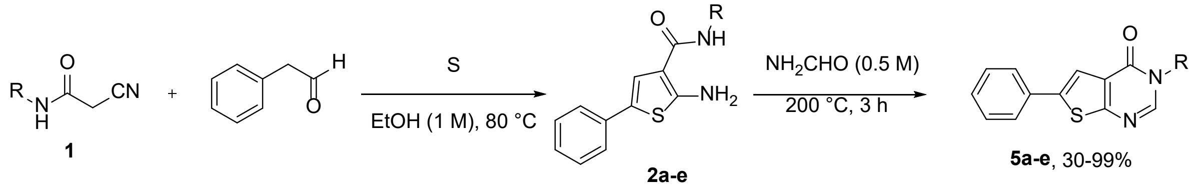

To a stirred solution of phenylacetaldehyde (1.0 mmol, 1.0 equiv) in EtOH (1.0 M), the corresponding cyanoacetamide (1.0 mmol, 1.0 equiv), sulfur (1.0 mmol, 1.0 equiv) and Et_3N (1 equiv, 1mmol) were added and the reaction mixture was stirred vigorously at 80 °C for 24 h. Then the reaction mixture was cooled down to rt. The solvent was removed under reduced pressure and the reaction mixture was diluted with ethyl acetate and extracted with water. The organic layer was collected and dried with sodium sulfate. The mixture was filtered and the solvent was removed under reduced pressure yielding the corresponding 2-aminothiophenes **2a**–**e**.

To a stirred solution of **2a**–**e** (0.8-1.0 mmol, 1.0 equiv), formamide (1.6–2.0 mmol, 2.0 equiv) was added and the reaction mixture was stirred vigorously at 200 °C for 3 h. Then the reaction mixture was cooled down to rt, diluted with ethyl acetate and extracted with water. The solvent was removed under reduced pressure and the reaction mixture was diluted with chloroform. Hexane of the same amount was added and the mixture was left at 4 °C for 24 h. The precipitate was filtered and washed with hexane to yield compounds **5a**–**e** in a total yield of 30–99% (2 steps).

General procedures for the synthesis of the 2-aminoquinolines ***3a*****–*****e***[3] *and pyrimidones* ***6a*****–*****e***

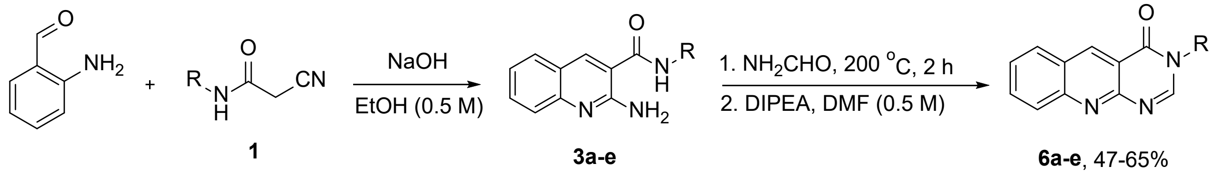

To a stirred solution of 2-aminobenzaldehyde (1.0 mmol, 1.0 equiv) in EtOH (0.5 M), the corresponding cyanoacetamide (1.0 mmol, 1.0 equiv) and NaOH (0.2 mmol, 0.2 equiv) were added and the reaction mixture was stirred vigorously at 70 °C for 10 min. Then, it was cooled down to 0 °C. The precipitate was filtered and washed with cold ethanol affording the 2-aminoquinolines **3a**–**e**.

To a stirred solution of **3a**–**e** (0.8–1.0 mmol, 1.0 equiv), formamide (1.6–2.0 mmol, 2.0 equiv) was added and the reaction mixture was stirred vigorously at 200 °C for 3 h. Then the reaction mixture was cooled down to rt, diluted with ethyl acetate and extracted with water. The solvent was removed under reduced pressure and the reaction mixture was diluted with chloroform. Hexane of the same amount was added and the mixture was left at 4 °C for 16 h. The precipitate was filtered and washed with hexane. To a stirred solution of **3a**–**e** (0.6–1.0 mmol, 1.0 equiv) in DMF (0.5 M), DIPEA (1.2–2.0 mmol, 2.0 equiv) was added and the reaction mixture was stirred vigorously at 160 °C for 12–16 h. Then the reaction mixture was cooled down to rt. The solvent was removed under reduced pressure and the reaction mixture was diluted with ethyl acetate and

extracted with water. The organic layer was collected and dried with sodium sulfate. The mixture was filtered and the solvent was removed under reduced pressure to yield compounds **6a**–**e** in a total yield of 47–65% (2 steps).

*General procedures for the synthesis of the 2-aminoindoles **4a**–**e**[4] and pyrimidones **7a**–**e***

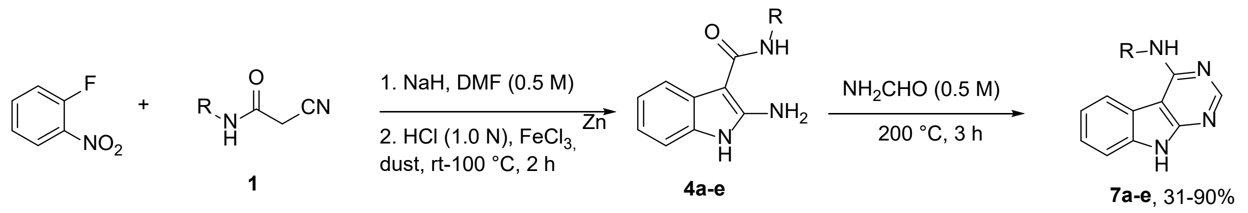

To a stirred solution of 1-fluoro-2-nitrobenzene (1.0 mmol, 1.0 equiv) in DMF (0.5 M), HCl (1.0 N, 2.0 mmol, 2 equiv) was added followed by the addition of iron trichloride (3.0–3.6 mmol, 3.0 equiv) and the Zn dust (10.0–12.0 mmol, 10 equiv) and the reaction mixture was stirred vigorously at 100 °C for 1 h. The reaction mixture was filtered in celite and the filtrate was extracted with water and sodium hydrogencarbonate. The organic layer was collected and dried with sodium sulfate. The mixture was filtrated and the solvent was removed under reduced pressure and the reaction mixture was diluted with chloroform. Hexane of the same amount was added and the mixture was left at 4 °C for 24 h. The precipitate was filtered and washed with hexane affording the 2-aminoindoles **4a**–**e**.

To a stirred solution of **4a**–**e** (0.8–1.0 mmol, 1.0 equiv), formamide (1.6–2.0 mmol, 2.0 equiv) was added and the reaction mixture was stirred vigorously at 200 °C for 3 h. Then the reaction mixture was cooled down to rt, diluted with ethyl acetate and extracted with water. The solvent was removed under reduced pressure and the reaction mixture was diluted with chloroform. Hexane of the same amount was added and the mixture was left at 4 °C for 24 h. The precipitate was filtered and washed with hexane to yield compounds **7a**–**e** in a total yield of 31–90% (2 steps).

3-Butyl-6-phenylthieno[2,3-*d*]pyrimidin-4(3*H*)-one (5a)

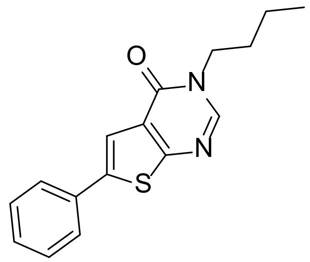

563 mg, 99% yield, brown solid. ^{1}H NMR (500 MHz, $CDCl_3$): 7.97 (s, 1H), 7.68 (s, 1H), 7.64 (d, J = 7 Hz, 2H), 7.42 (t, J = 7 Hz, 2H), 7.36-7.33 (m, 1H), 4.02 (t, J = 7.5 Hz, 2H), 1.79 (quint, J = 7.5 Hz, 2H), 1.42 (sext, J = 7.5 Hz, 2H), 0.98 (t, J = 7.5 Hz, 3H); ^{13}C NMR (125 MHz, $CDCl_3$): δ 163.0, 157.6, 146.4, 142.2, 133.5, 129.2, 128.7, 126.3, 126.1, 117.3, 46.9, 31.7, 20.0, 13.7; HRMS (ESI) m/z: $[M-H]^-$: $C_{16}H_{16}N_2OS$-H, calculated 283.091056; found 283.26413.

3-Cyclohexyl-6-phenylthieno[2,3-*d*]pyrimidin-4(3*H*)-one (5b)

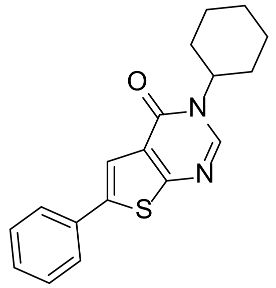

324 mg, 70% yield, brown solid. ^{1}H NMR (500 MHz, $CDCl_3$): 8.05 (s, 1H), 7.68 (s, 1H), 7.65 (d, J = 7 Hz, 2H), 7.42 (t, J = 7 Hz, 2H), 7.34 (t, J = 7 Hz, 1H), 4.85 (tt, J_1 = 12 Hz, J_2 = 3.5 Hz, 1H), 2.04-2.02 (m, 2H), 1.96-1.93 (m, 2H), 1.81-1.78 (m, 1H), 1.65-1.49 (m, 4H), 1.36-1.21 (m, 1H); ^{13}C NMR (125 MHz, $CDCl_3$): δ 162.4, 157.1, 143.8, 141.8, 133.4, 129.1, 128.5, 126.1, 125.5, 117.3, 53.2, 33.0, 25.9, 25.2; HRMS (ESI) m/z: $[M-H]^-$: $C_{18}H_{18}N_2OS$-H, calculated 309.106706; found 309.17206.

3-(Naphthalen-2-ylmethyl)-6-phenylthieno[2,3-*d*]pyrimidin-4(3*H*)-one (5c)

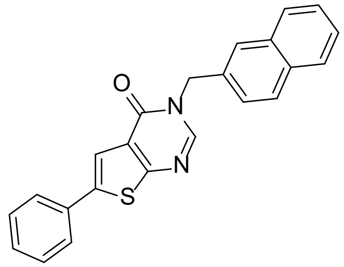

105 mg, 30% yield, brown solid. ^{1}H NMR (500 MHz, $CDCl_3$): 8.01 (d, J = 8.5 Hz, 1H), 7.91-7.88 (m, 3H), 7.76 (s, 1H), 7.66-7.64 (m, 2H), 7.58-7.52 (m, 3H), 7.49-7.42 (m, 2H), 7.39-7.36 (m, 2H), 5.69 (s, 2H); ^{13}C NMR (125 MHz, $CDCl_3$): δ 162.9, 157.6, 145.9, 142.5, 134.1, 133.4, 131.1, 130.9,

129.7, 129.3, 129.2, 128.8, 127.4, 127.4, 126.6, 126.3, 125.8, 125.5, 123.1, 117.4, 46.7; HRMS (ESI) m/z: $[M-H]^-$: $C_{23}H_{16}N_2OS$-H, calculated 367.091056; found 367.03128.

3-(Furan-2-ylmethyl)-6-phenylthieno[2,3-*d*]pyrimidin-4(3*H*)-one (5d)

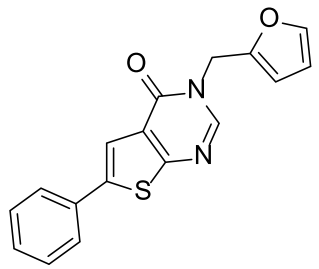

106 mg, 38% yield, brown solid. ^{1}H NMR (500 MHz, $CDCl_3$): 8.12 (s, 1H), 7.68 (s, 1H), 7.63 (d, *J* = 7 Hz, 2H), 7.43-7.39 (m, 3H), 7.36-7.33 (m, 1H), 6.49 (dd, J_1 = 3.5 Hz, J_2 = 1 Hz, 1H), 6.36 (dd, J_1 = 3.5 Hz, J_2 = 2 Hz, 1H), 5.21 (s, 1H); ^{13}C NMR (125 MHz, $CDCl_3$): δ 162.8, 157.0, 148.1, 145.9, 143.3, 142.3, 133.2, 129.1, 128.6, 126.1, 125.8, 117.1, 110.8, 110.3, 41.9; HRMS (ESI) m/z: $[M-H]^-$: $C_{17}H_{12}N_2O_2S$-H, calculated 307.054671; found 306.91909.

6-Phenyl-3-(thiophen-2-ylmethyl)thieno[2,3-*d*]pyrimidin-4(3*H*)-one (5e)

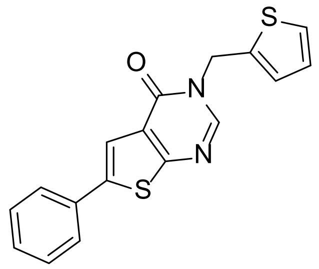

144 mg, 45% yield, brown solid. ^{1}H NMR (500 MHz, $CDCl_3$): 8.09 (s, 1H), 7.70 (s, 1H), 7.63 (d, *J* = 7 Hz, 2H), 7.42 (t, *J* = 7 Hz, 2H), 7.37-7.33 (m, 1H), 7.29 (dd, J_1 = 5 Hz, J_2 = 1.5 Hz, 1H), 7.17 (dd, J_1 = 3.5 Hz, J_2 = 1 Hz, 1H), 6.98 (dd, J_1 = 5 Hz, J_2 = 3.5 Hz, 1H), 5.37 (s, 1H); ^{13}C NMR (125 MHz, $CDCl_3$): δ 162.8, 157.1, 145.6, 142.4, 137.3, 133.2, 129.1, 128.7, 128.0, 127.1, 126.8, 126.1, 125.8, 117.1, 44.2; HRMS (ESI) m/z: $[M-H]^-$: $C_{17}H_{12}N_2OS_2$-H, calculated 323.031828; found 323.11362.

3-Butylpyrimido[4,5-*b*]quinolin-4(3*H*)-one (6a)

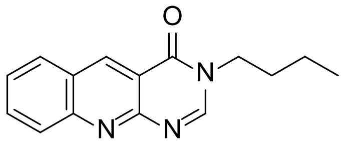

82 mg, 65% yield, brown solid. ^{1}H NMR (500 MHz, DMSO): 9.34 (s, 1H), 8.66 (s, 1H), 8.29 (dd, J_1 = 8.5 Hz, J_2 = 1.5 Hz, 1H), 8.10 (dd, J_1 = 8.5 Hz, J_2 = 1 Hz, 1H), 7.97-7.94 (m, 1H), 7.70-7.67 (m, 1H), 4.01 (t, *J* = 7.5 Hz, 2H), 1.71 (quint, *J* = 7.5 Hz, 2H), 1.35 (sext, *J* = 7.5 Hz, 2H), 0.93 (t, *J* = 7.5 Hz, 3H); ^{13}C NMR (125 MHz, DMSO): δ 161.1, 155.6, 151.9, 150.6, 138.6, 132.9, 129.5, 128.5, 126.7, 126.3, 116.1, 45.7, 30.7, 19.3, 13.6; HRMS (ESI) m/z: $[M+H]^+$: $C_{15}H_{15}N_3O$+H, calculated 254.128791; found 254.12789

3-(Furan-2-ylmethyl)pyrimido[4,5-*b*]quinolin-4(3*H*)-one (6b)

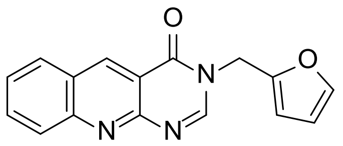

128 mg, 47% yield, brown solid. ^{1}H NMR (500 MHz, DMSO): 9.35 (s, 1H), 8.74 (s, 1H), 8.30 (dd, J_1 = 8 Hz, J_2 = 1.5 Hz), 8.10 (d, *J* = 8.5 Hz), 7.98-7.95 (m, 1H), 7.71-7.68 (m, 1H), 7.64 (d, *J* = 2 Hz, 1H), 6.53 (d, *J* = 3 Hz, 1H), 6.45-6.44 (m, 1H), 5.26 (s, 2H); ^{13}C NMR (125 MHz, DMSO): δ 160.7, 155.4, 151.3, 150.6, 149.3, 143.3, 138.9, 133.1, 129.6, 128.5, 126.9, 126.4, 116.1, 110.8, 109.1, 41.8; HRMS (ESI) m/z: $[M+H]^+$: $C_{15}H_{15}N_3O$+H, calculated 278.09240; found 278.09129.

3-(3-Ethoxypropyl)pyrimido[4,5-*b*]quinolin-4(3*H*)-one (6c)

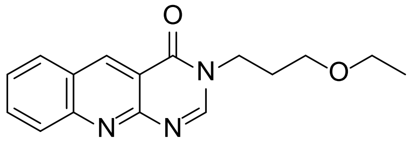

144 mg, 51% yield, brown solid. ^{1}H NMR (500 MHz, DMSO): 9.31 (s, 1H), 8.59 (s, 1H), 8.29 (dd, J_1 = 8.5 Hz, J_2 = 1.5 Hz, 1H), 8.09 (d, *J* = 9 Hz, 1H), 7.96-7.93 (m, 1H), 7.69-7.66 (m, 1H), 4.08 (t, *J* = 7 Hz, 2H), 3.44 (t, *J* = 6 Hz, 2H), 3.36 (q, *J* = 7 Hz, 2H), 1.97 (quint, *J* = 6.5 Hz, 2H), 1.01 (t, *J* = 7 Hz, 3H); ^{13}C NMR (125 MHz, DMSO): δ 161.2, 155.6, 152.0, 150.6, 138.5, 132.9, 129.5, 128.5, 126.6, 126.3, 116.1, 67.1, 65.3, 44.1, 28.3, 15.0; HRMS (ESI) m/z: $[M+H]^+$: $C_{16}H_{17}N_3O_2$+H, calculated 284.13935; found 284.13834.

3-(3,3-Diphenylpropyl)pyrimido[4,5-*b*]quinolin-4(3*H*)-one (6d)

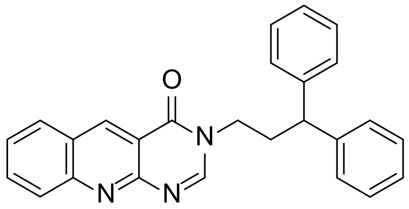

203 mg, 52% yield, brown solid. ^{1}H NMR (500 MHz, DMSO): 9.30 (s, 1H), 8.48 (s, 1H), 8.28 (dd, J_1 = 8.5 Hz, J_2 = 1.5 Hz, 1H), 8.09 (d, *J* = 8.5 Hz, 1H), 7.97-7.93 (m, 1H), 7.70-7.67 (m, 1H), 7.36 (d, *J* = 7.5 Hz, 4H), 7.26 (t, *J* = 7.5 Hz, 4H), 7.12 (t, *J* = 7.5 Hz, 2H), 4.11 (t, *J* = 7.5 Hz, 1H), 3.96 (t, *J* = 7.5 Hz, 2H), 2.56 (q, *J* = 7.5 Hz, 2H); ^{13}C NMR (125 MHz, DMSO): δ 161.6, 156.0, 152.2, 151.0, 144.7, 139.0, 133.3, 130.0, 128.9, 128.0, 127.1, 126.7, 126.7, 116.5, 48.8, 45.7, 33.9; HRMS (ESI) m/z: $[M+H]^+$: $C_{26}H_{21}N_3O$+H, calculated 392.17574; found 392.17440.

3-Cyclohexylpyrimido[4,5-*b*]quinolin-4(3*H*)-one (6e)

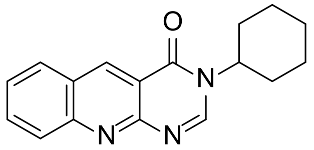

153 mg, 55% yield, brown solid. ^{1}H NMR (500 MHz, DMSO): 9.34 (s, 1H), 8.74 (s, 1H), 8.30 (d, *J* = 8.5 Hz, 1H), 8.10 (d, *J* = 8.5 Hz, 1H), 7.97-7.93 (m, 1H), 7.69-7.66 (m, 1H), 4.65-4.59 (m, 1H), 1.88-1.87 (m, 6H), 1.70-1.66 (m, 1H), 1.46-1.43 (m, 2H), 1.29-1.22 (m, 1H); ^{13}C NMR (125 MHz, DMSO): δ 160.8, 155.1, 150.7, 149.5, 138.9, 132.9, 129.5, 128.5, 126.6, 126.4, 115.9, 53.5, 31.1, 25.6, 24.7; HRMS (ESI) m/z: $[M+H]^+$: $C_{17}H_{17}N_3O$+H, calculated 280.14444; found 280.14340.

3-Benzyl-3,9-dihydro-4*H*-pyrimido[4,5-*b*]indol-4-one (7a)

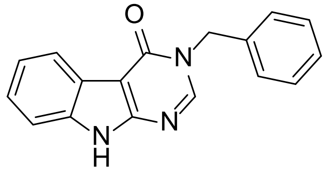

51 mg, 38% yield, brown solid. ^{1}H NMR (500 MHz, DMSO) δ 12.25 (s, 1H), 8.64 (s, 1H), 7.99 (d, *J* = 8 Hz, 1H), 7.48 (d, *J* = 8.5 Hz, 1H), 7.37-7.32 (m, 5H), 7.30-7.23 (m, 2H), 5.27 (s, 2H); ^{13}C NMR (125 MHz, DMSO) δ 157.3, 153.0, 150.1, 137.5, 135.7, 128.6, 127.6, 127.6, 124.4, 122.0, 121.2, 120.6, 111.7, 99.5, 48.2; HRMS (ESI) m/z: $[M-H]^-$: $C_{17}H_{13}N_3O$-H, calculated 274.098583; found 273.950011.

3-Butyl-3,9-dihydro-4*H*-pyrimido[4,5-*b*]indol-4-one (7b)

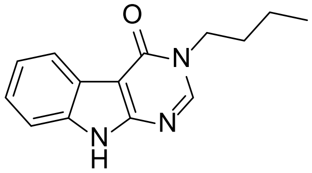

218 mg, 90% yield, white solid. ^{1}H NMR (500 MHz, DMSO): 12.16 (s, 1H), 8.45 (s, 1H), 8.01 (d, *J* = 8 Hz, 1H), 7.47 (d, *J* = 8 Hz, 1H), 7.35-7.32 (m, 1H), 7.26-7.22 (m, 1H), 4.04 (t, *J* = 7.5 Hz, 2H), 1.69 (quint, *J* = 7.5 Hz, 2H), 1.32 (q, *J* = 7.5 Hz, 2H), 0.92 (t, *J* = 7.5 Hz, 3H); ^{13}C NMR (125 MHz, DMSO): δ 162.9, 157.4, 153.0, 150.0, 135.7, 124.2, 122.0, 121.1, 120.6, 111.6, 99.3, 45.1, 31.4, 19.3, 13.6; HRMS (ESI) m/z: $[M-H]^-$: $C_{14}H_{15}N_3O$-H, calculated 242.12879; found 242.12789.

3-(2-Chlorobenzyl)-3,9-dihydro-4*H*-pyrimido[4,5-*b*]indol-4-one (7c)

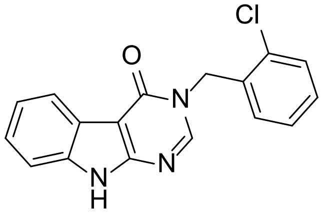

114 mg, 31% yield, yellow solid. ^{1}H NMR (500 MHz, DMSO): 12.32 (s, 1H), 8.57 (s, 1H), 7.98 (d, *J* = 8 Hz, 1H), 7.52-7.50 (m, 2H), 7.37-7.31 (m, 2H), 7.30-7.23 (m, 2H), 6.99 (dd, J_1 = 8 Hz, J_2 = 1.5 Hz, 1H), 5.34 (s, 1H); ^{13}C NMR (125 MHz, DMSO): δ 157.3, 153.1, 150.5, 135.8, 134.5, 131.8, 129.4, 129.2, 128.3, 127.5, 124.5, 122.0, 121.3, 120.6, 111.8, 99.4, 46.6; HRMS (ESI) m/z: $[M+H]^+$: $C_{17}H_{12}ClN_3O$+H, calculated 308,059611; found 308,97190.

3-(4-Chlorobenzyl)-3,9-dihydro-4*H*-pyrimido[4,5-*b*]indol-4-one (7d)

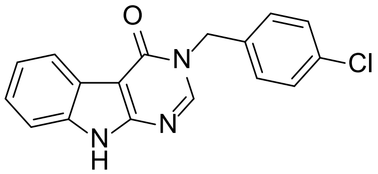

117 mg, 35% yield, yellow solid. ^{1}H NMR (500 MHz, DMSO) δ 12.26 (s, 1H), 8.65 (s, 1H), 7.99 (d, *J* = 7.5 Hz, 1H), 7.49 (d, *J* = 8 Hz, 1H), 7.40 (s, 4H), 7.36-7.33 (m, 1H), 7.24 (dd, J_1 = 8 Hz, J_2 = 1.5 Hz, 1H), 5.25 (s, 2H); ^{13}C NMR (125 MHz, DMSO) δ 157.3, 153.0, 150.1, 136.5, 135.7, 132.3, 129.7, 128.6, 124.4, 122.0, 121.3, 120.6, 111.8, 99.5, 47.8; HRMS (ESI) m/z: $[M-H]^-$: $C_{17}H_{12}ClN_3O$-H, calculated 308.059611; found 308.05953.

3-Cyclohexyl-3,9-dihydro-4*H*-pyrimido[4,5-*b*]indol-4-one (7e)

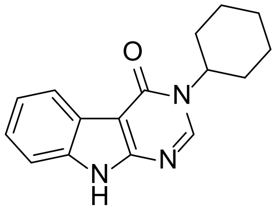

91 mg, 34% yield, yellow solid. ^{1}H NMR (500 MHz, DMSO) δ 12.16 (s, 1H), 8.49 (s, 1H), 8.01 (d, *J* = 8 Hz, 1H), 7.47 (d, J_1 = 8 Hz, 1H), 7.35-7.32 (m, 1H), 7.26-7.23 (m, 1H), 4.78-4.72 (m, 1H), 1.88-1.80 (m, 6H), 1.71-1.69 (m, 1H), 1.47-1.39 (m, 2H), 1.29-1.23 (m, 1H); ^{13}C NMR (125 MHz, DMSO) δ 157.6, 144.4, 128.6, 127.5, 126.3, 122.1, 88.7, 70.0, 32.1, 26.8; HRMS (ESI) m/z: [M+H]$^+$: $C_{16}H_{17}N_3O$+H, calculated 266.129883; found 266.12988.

3. Exemplary copies of NMR spectra of novel compounds

3-Butyl-6-phenylthieno[2,3-*d*]pyrimidin-4(3*H*)-one (5a)

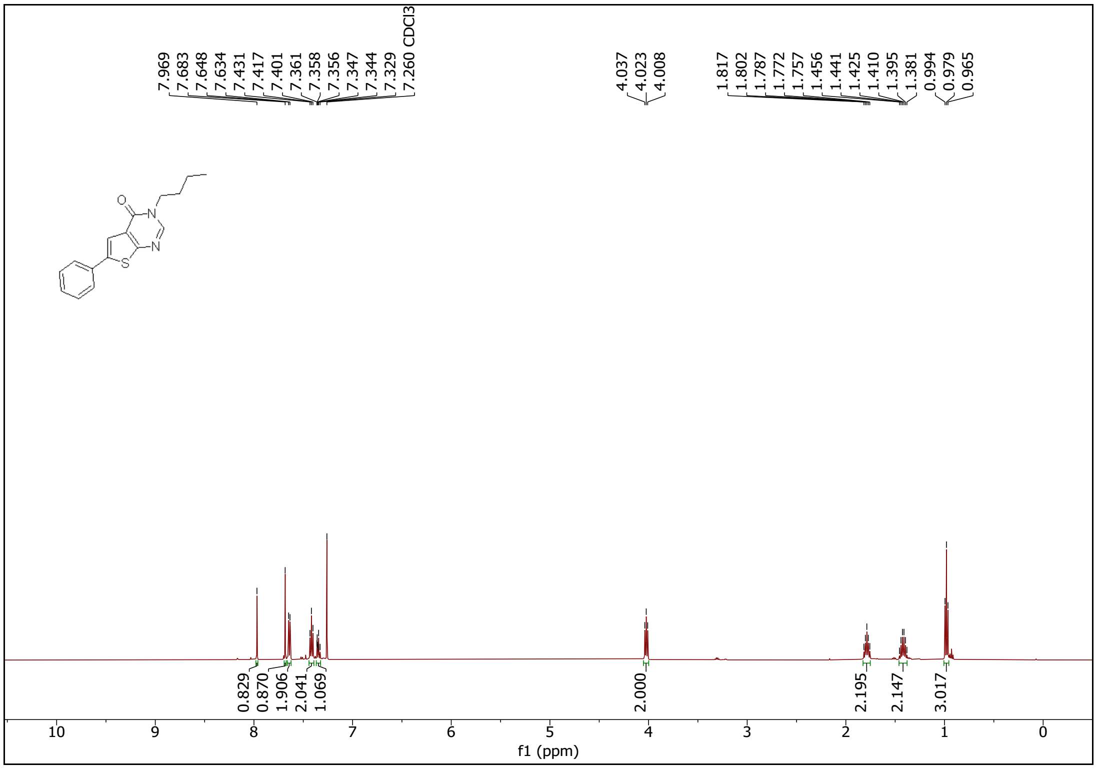

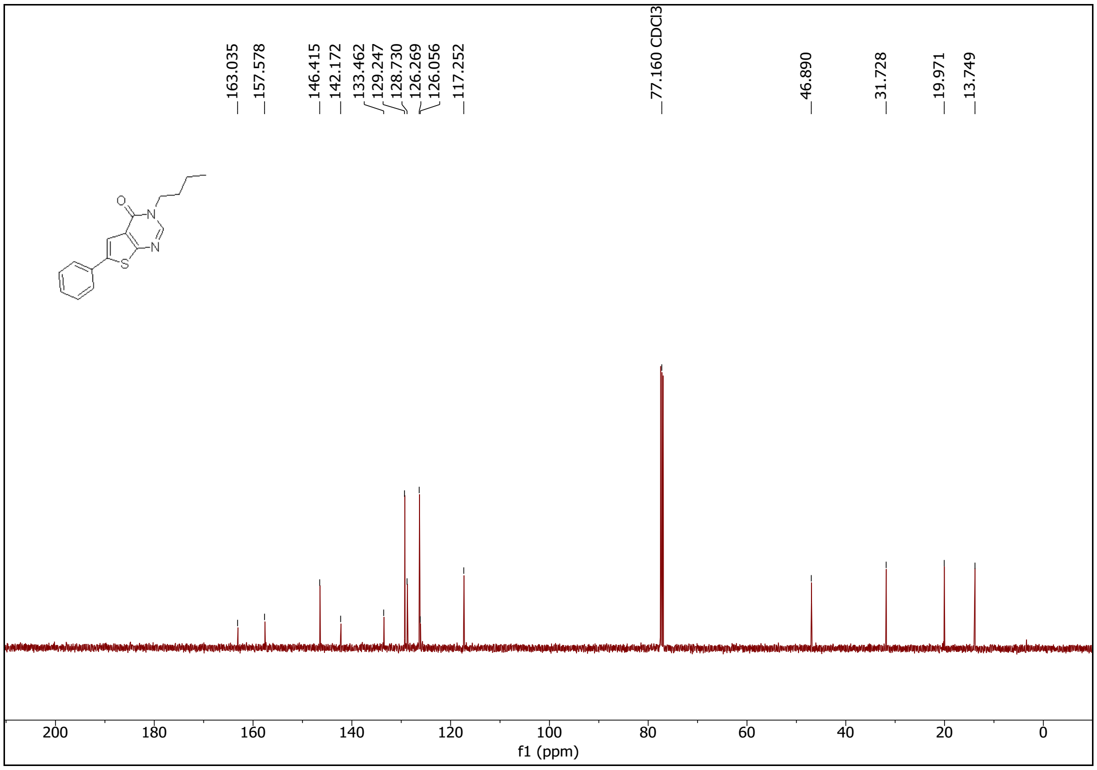

3-Cyclohexyl-6-phenylthieno[2,3-*d*]pyrimidin-4(3*H*)-one (5b)

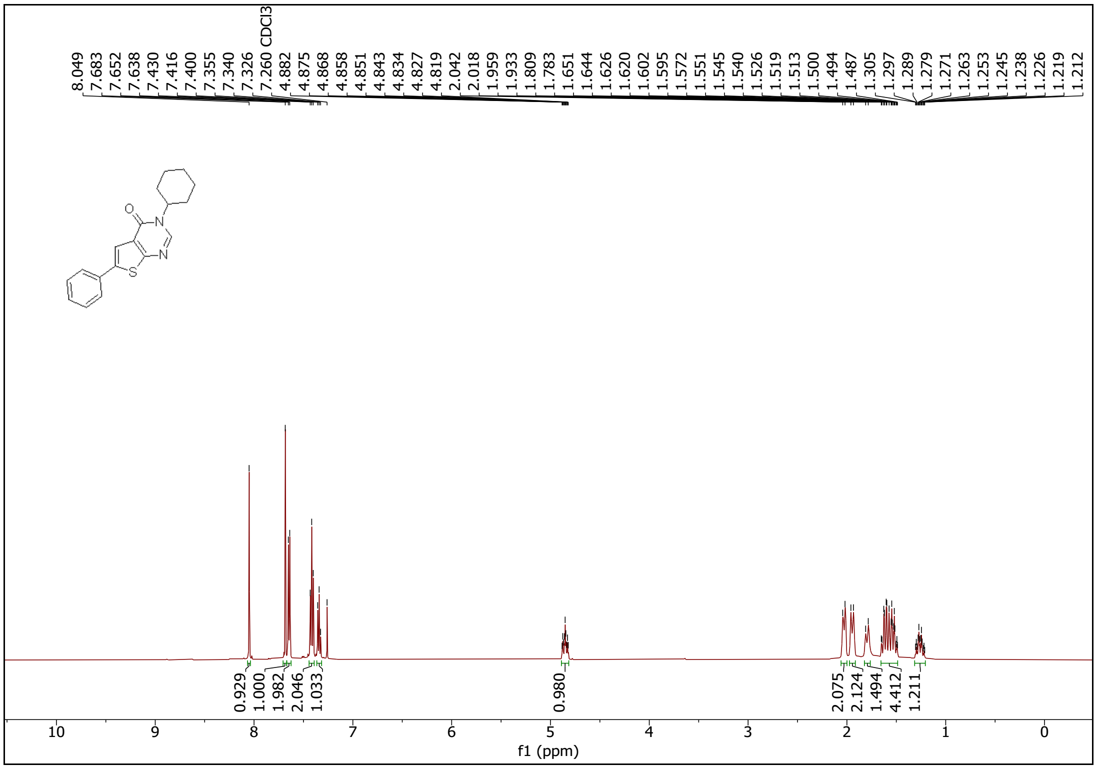

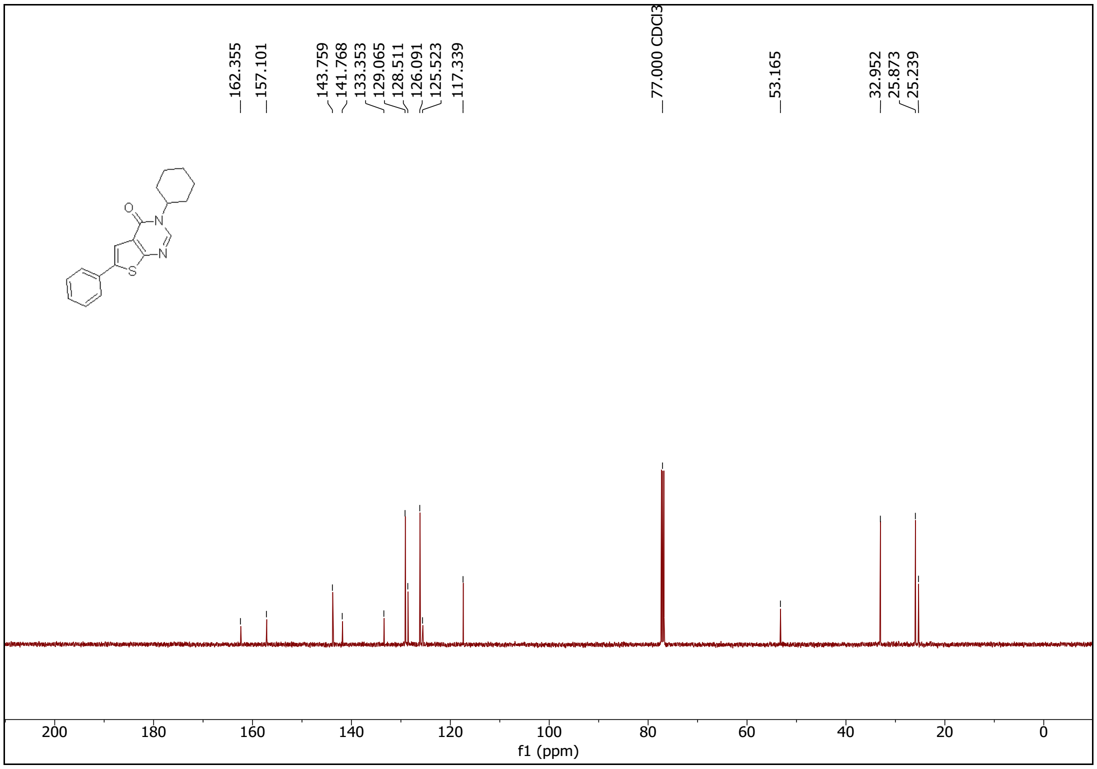

3-(Naphthalen-2-ylmethyl)-6-phenylthieno[2,3-*d*]pyrimidin-4(3*H*)-one (5c)

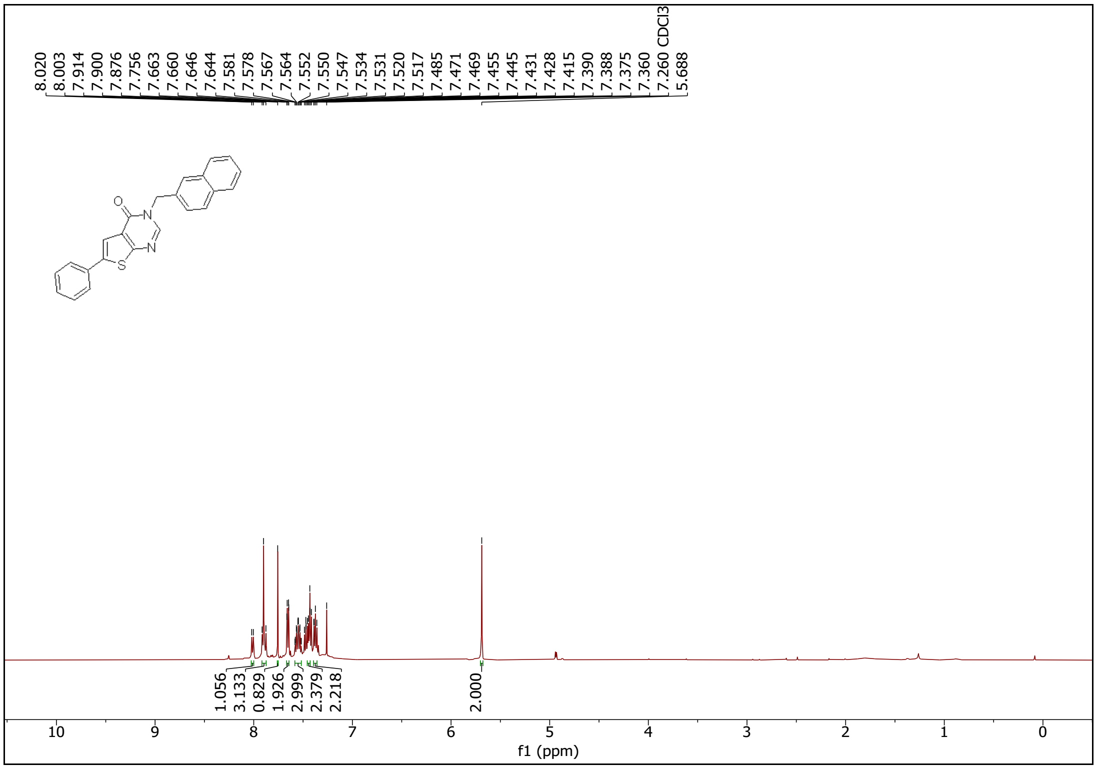

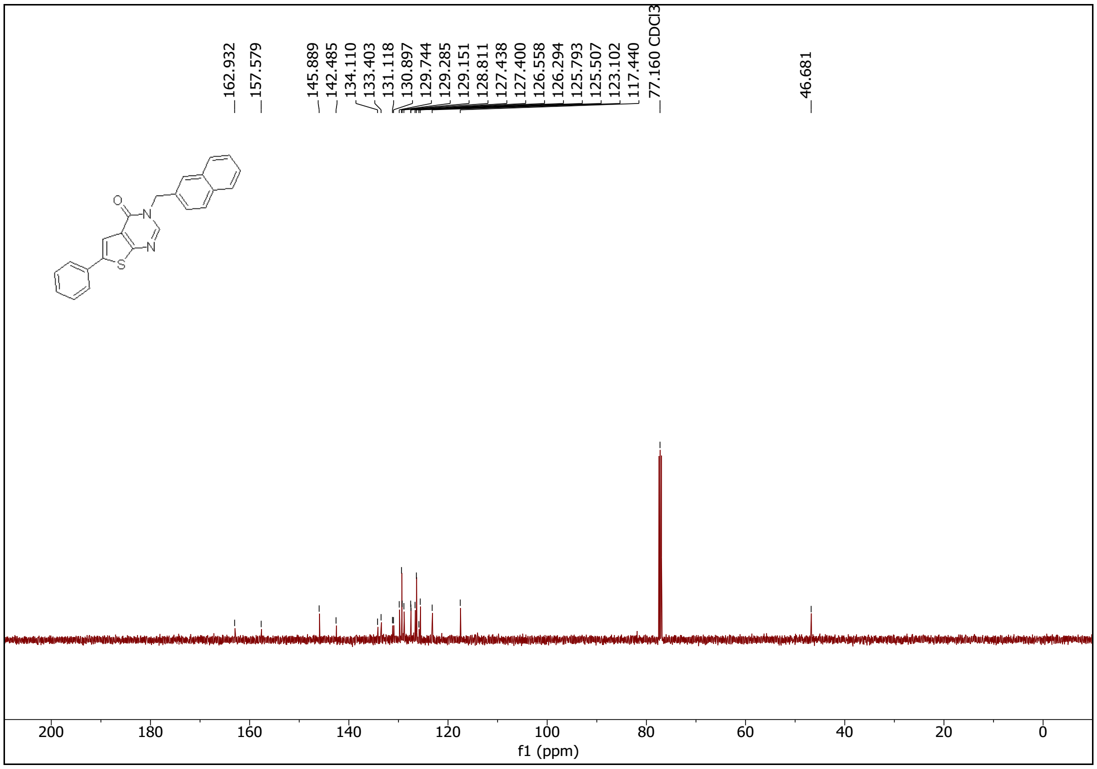

3-(Furan-2-ylmethyl)-6-phenylthieno[2,3-*d*]pyrimidin-4(3*H*)-one (5d)

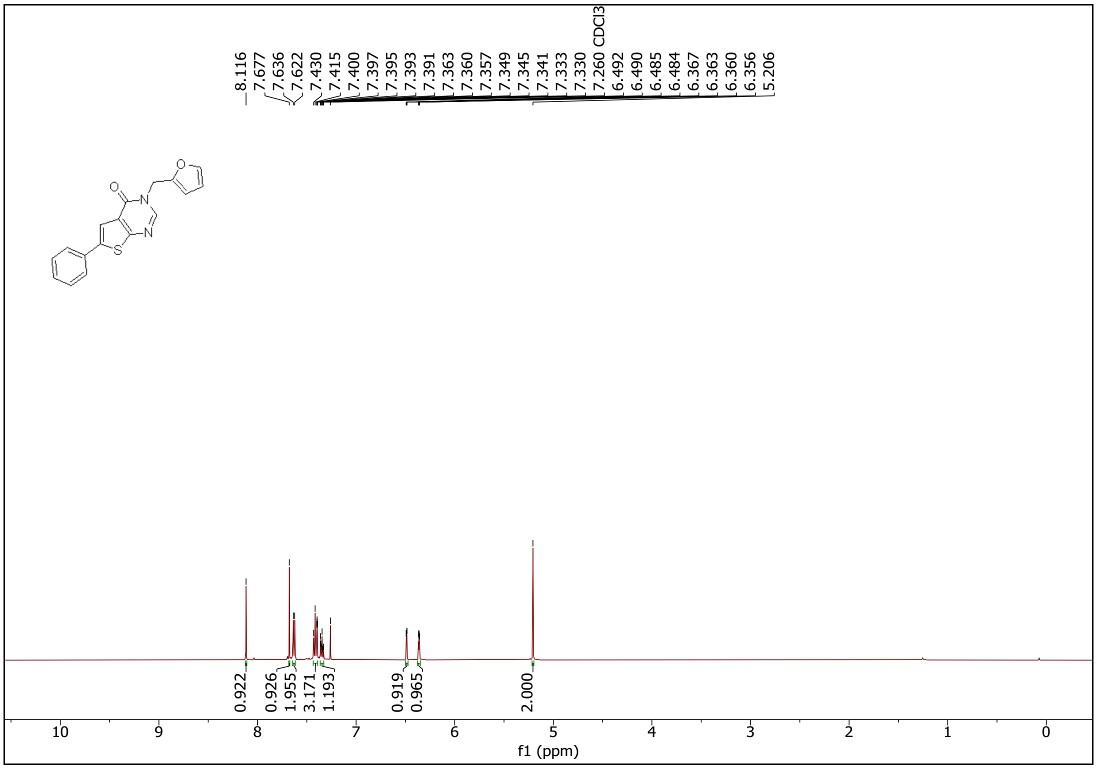

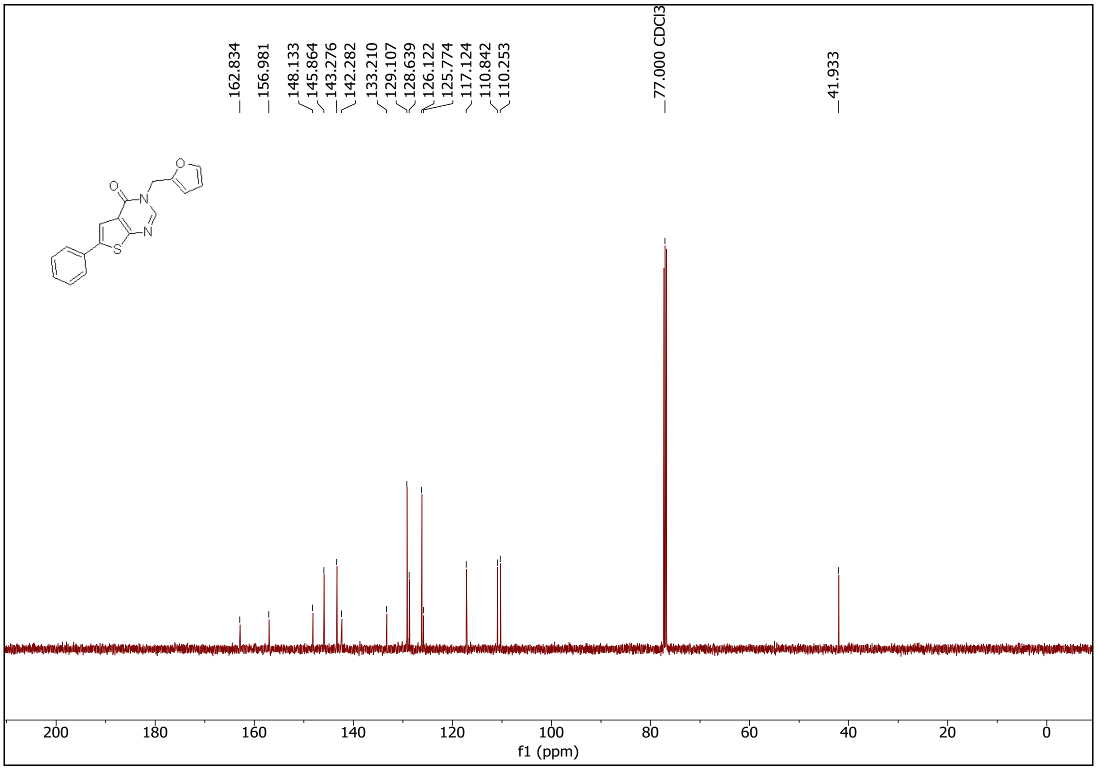

6-Phenyl-3-(thiophen-2-ylmethyl)thieno[2,3-*d*]pyrimidin-4(3*H*)-one (5e)

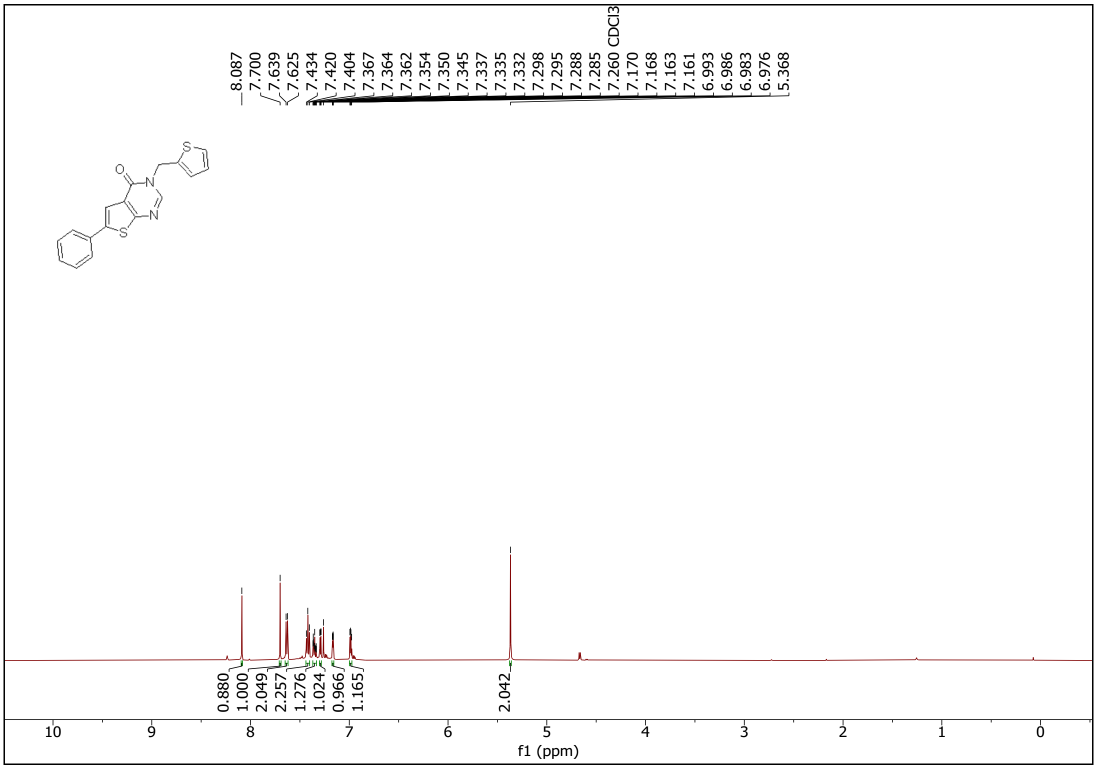

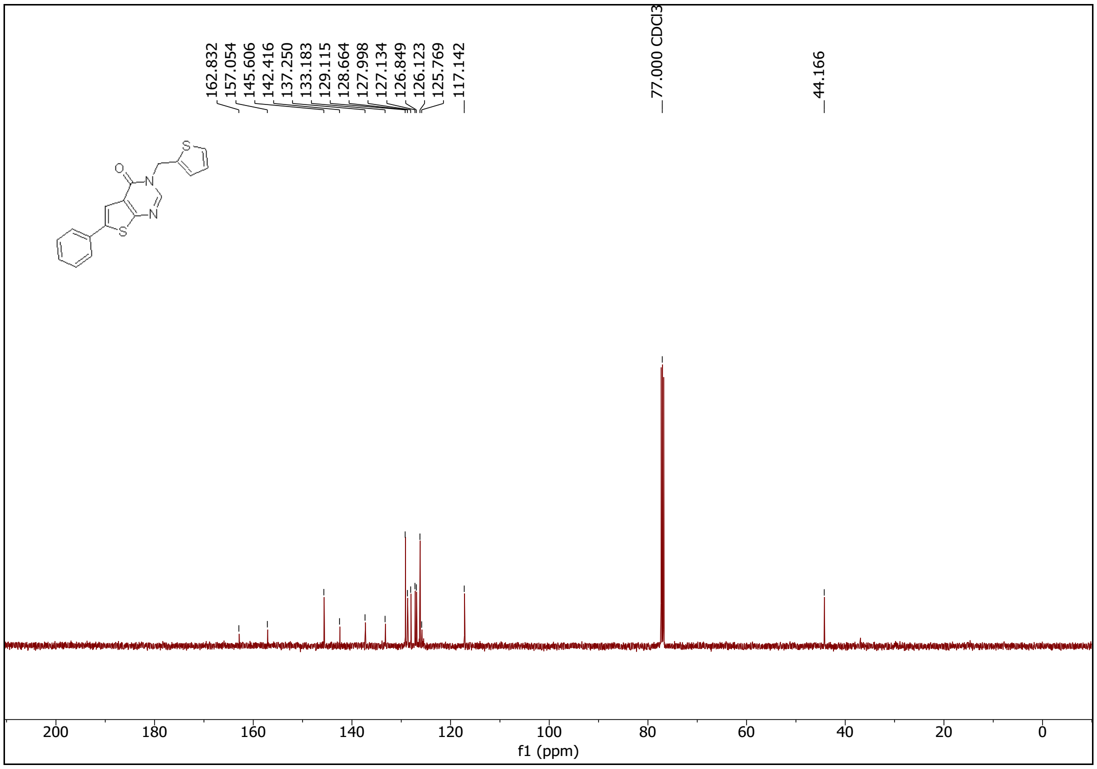

3-Butylpyrimido[4,5-*b*]quinolin-4(3*H*)-one (6a)

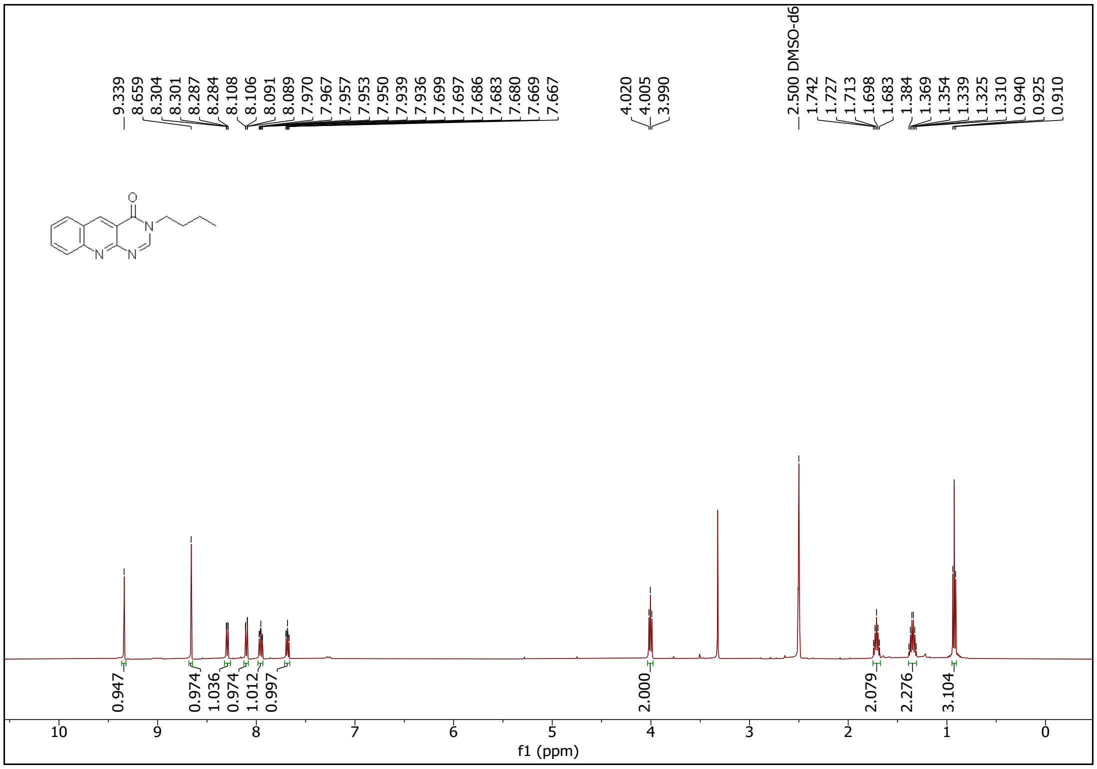

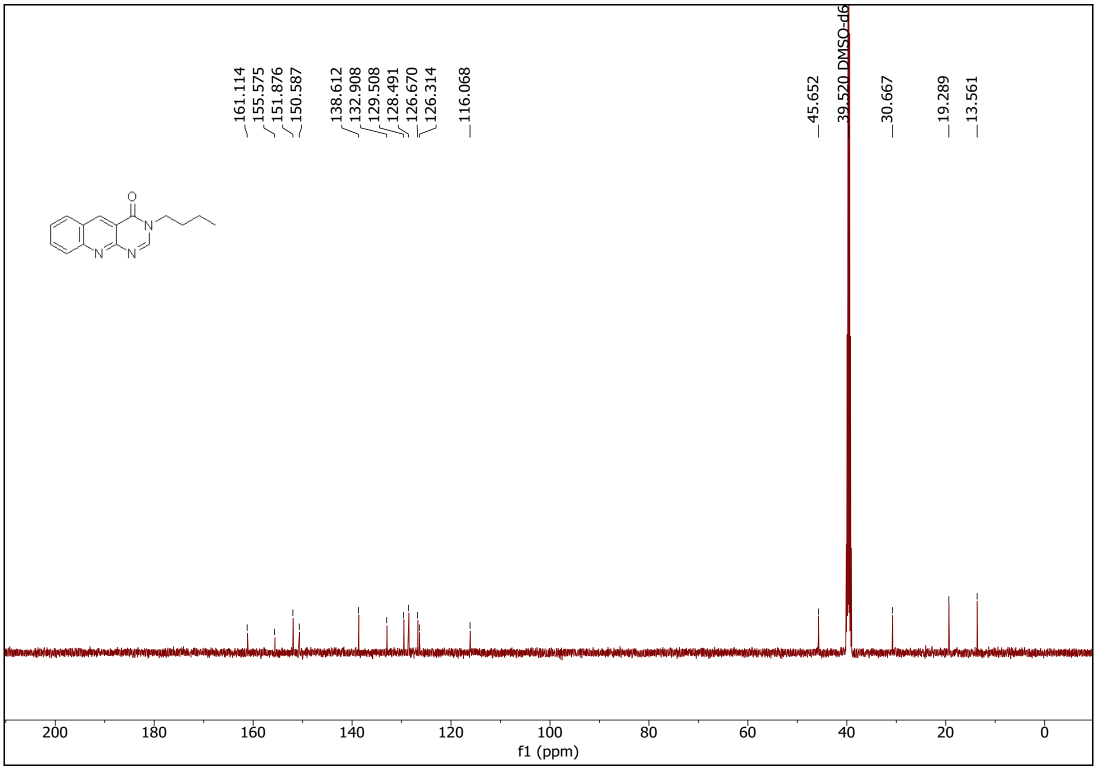

3-(Furan-2-ylmethyl)pyrimido[4,5-*b*]quinolin-4(3*H*)-one (6b)

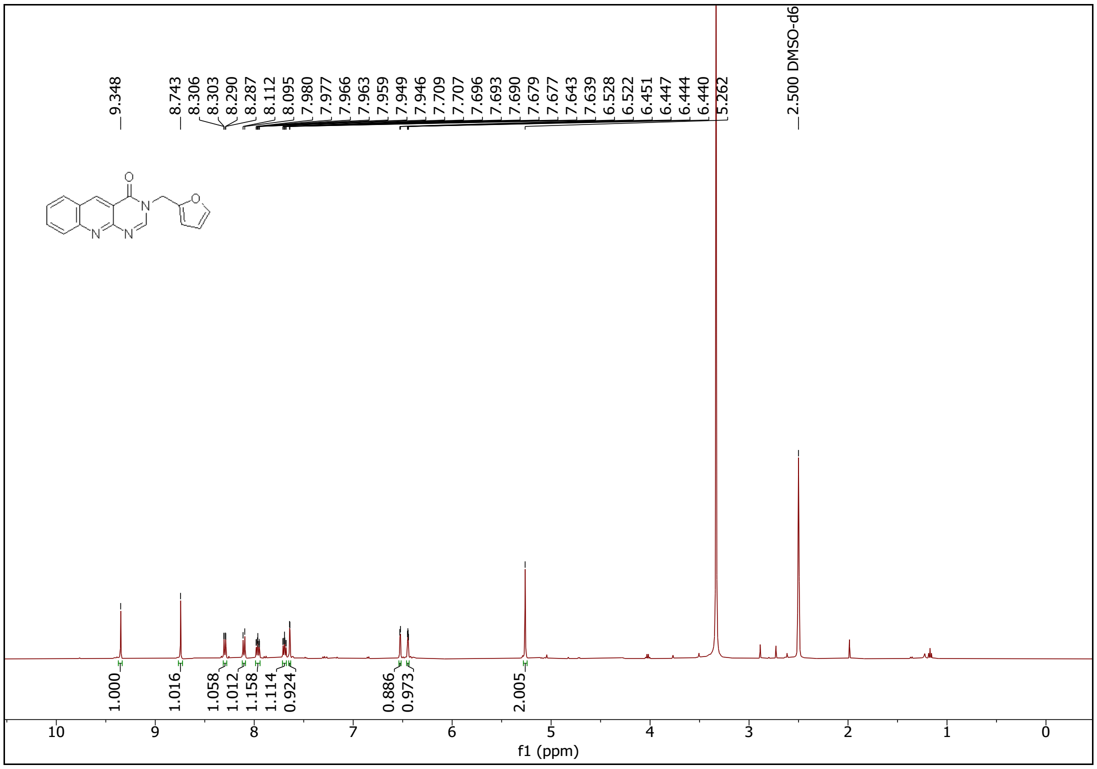

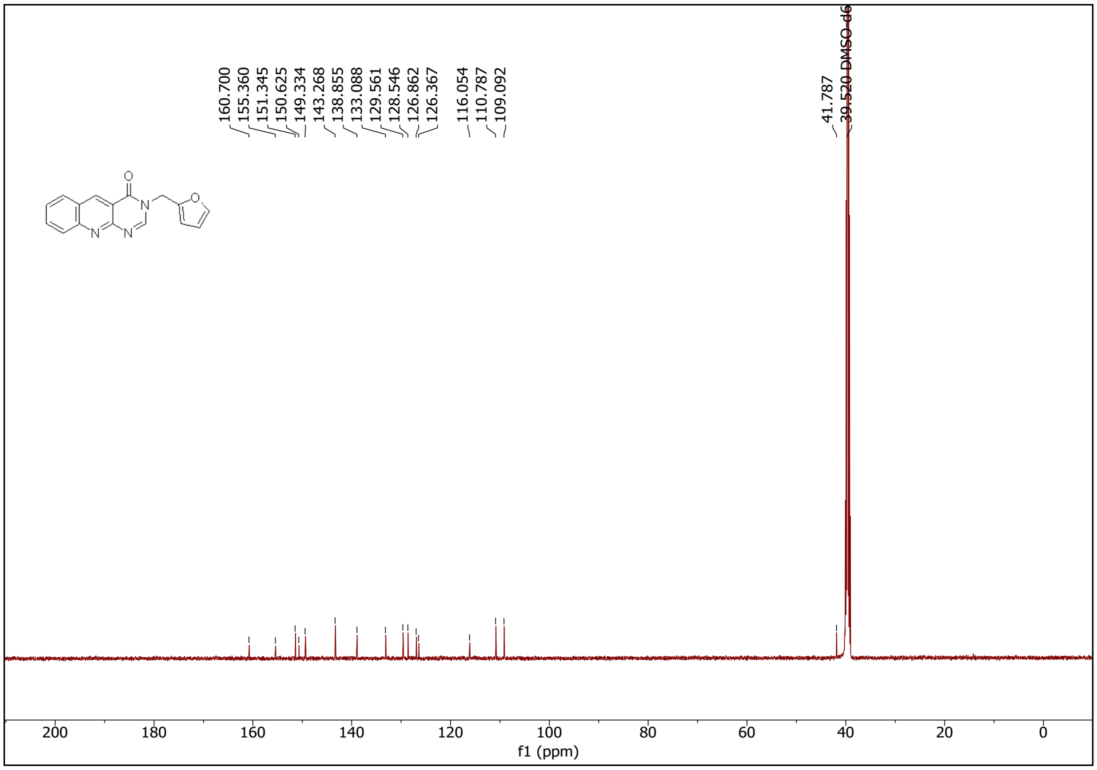

3-(3-Ethoxypropyl)pyrimido[4,5-*b*]quinolin-4(3*H*)-one (6c)

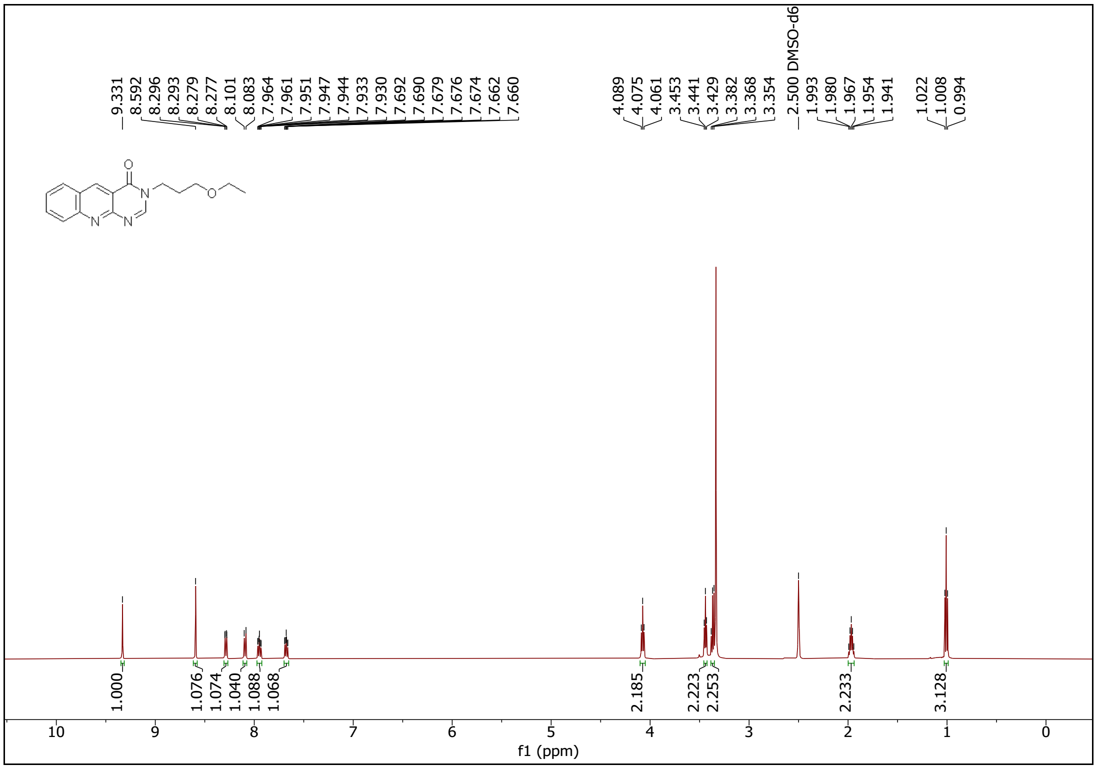

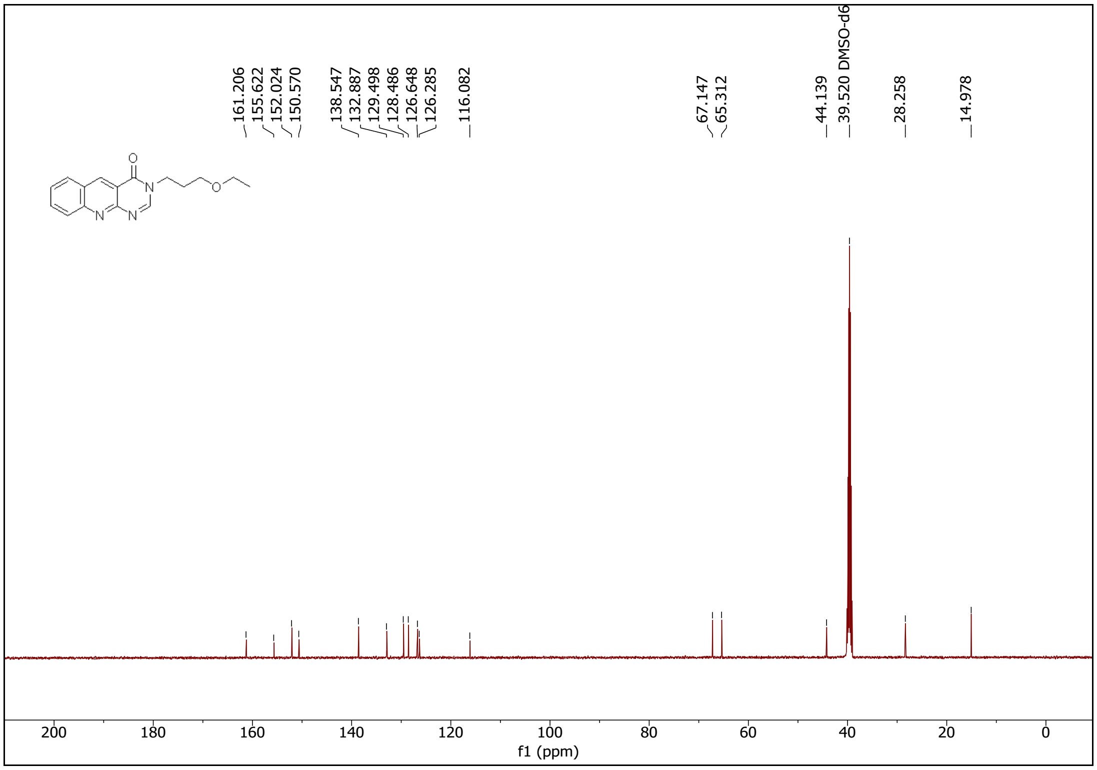

HMBC

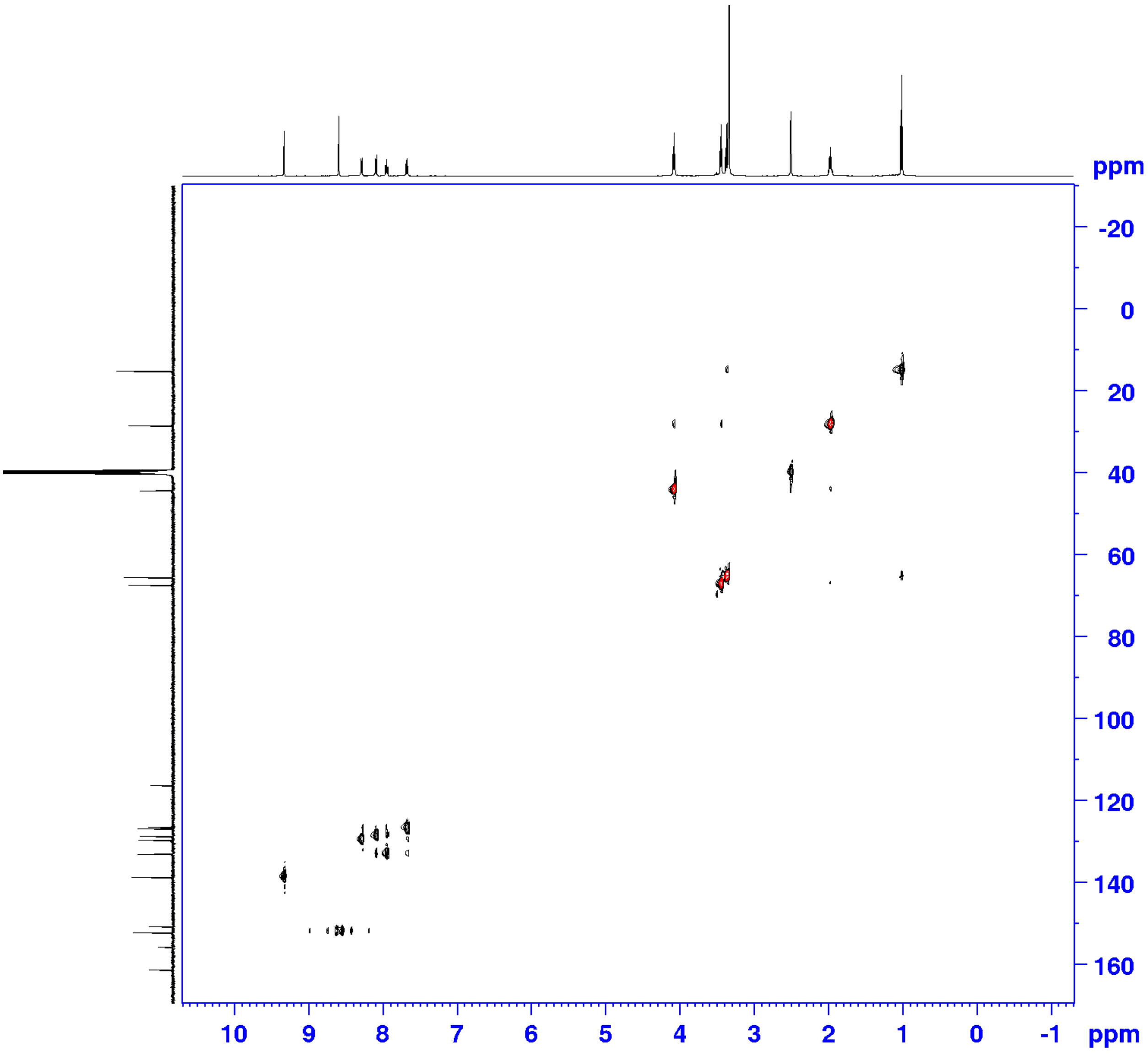

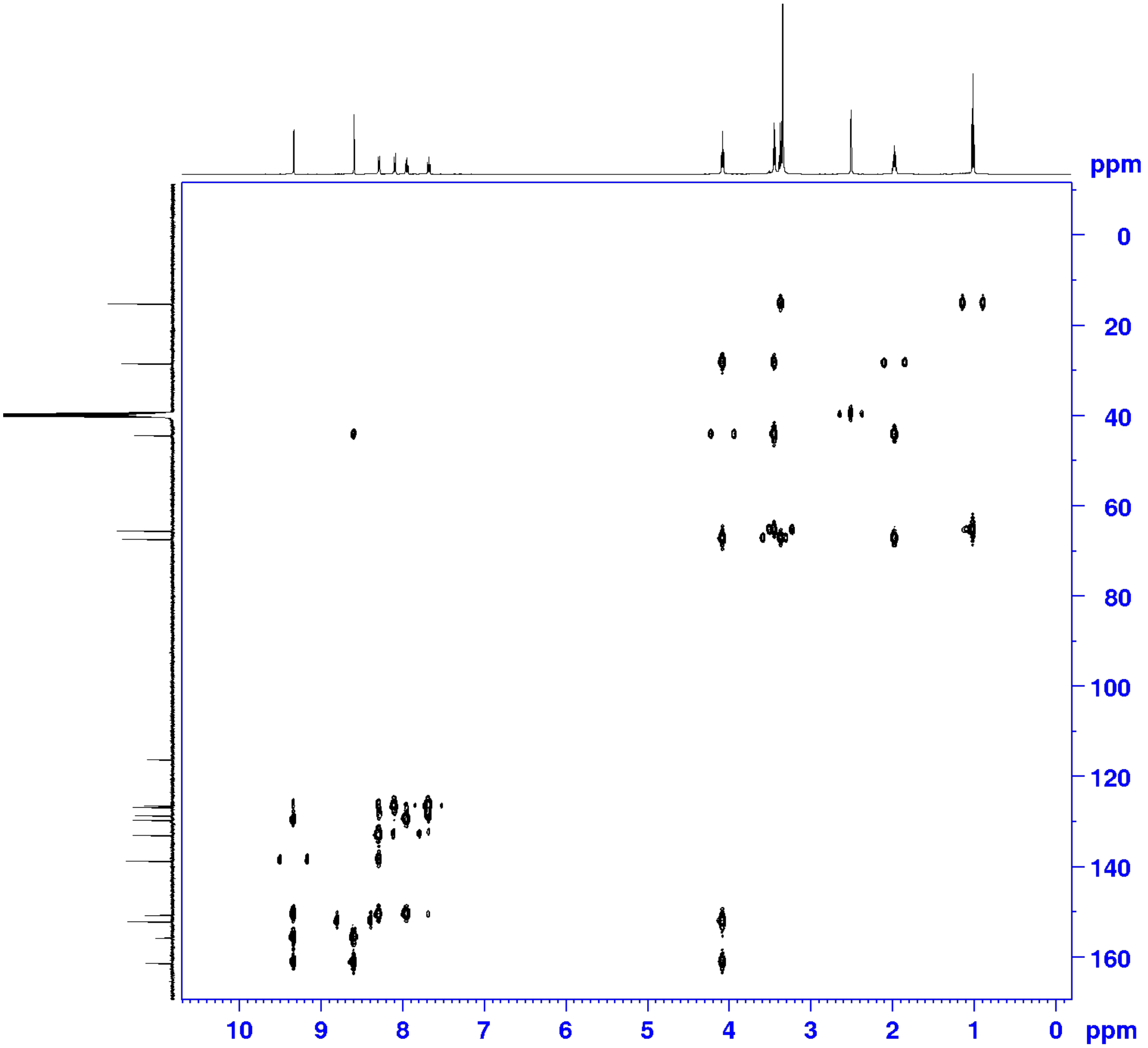
ppm
0
20
40
60
80
100
120
140
160
10
9
8
7
6
5
4
3
2
1
0
ppm

3-(3,3-Diphenylpropyl)pyrimido[4,5-*b*]quinolin-4(3*H*)-one (6d)

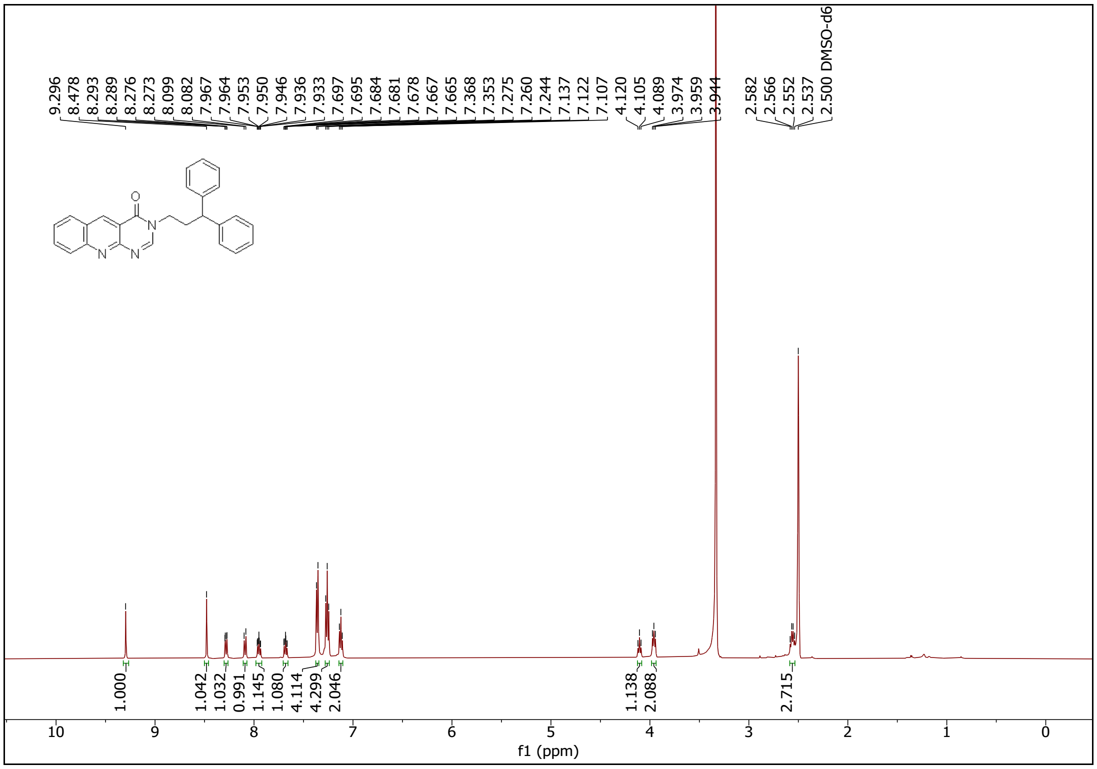

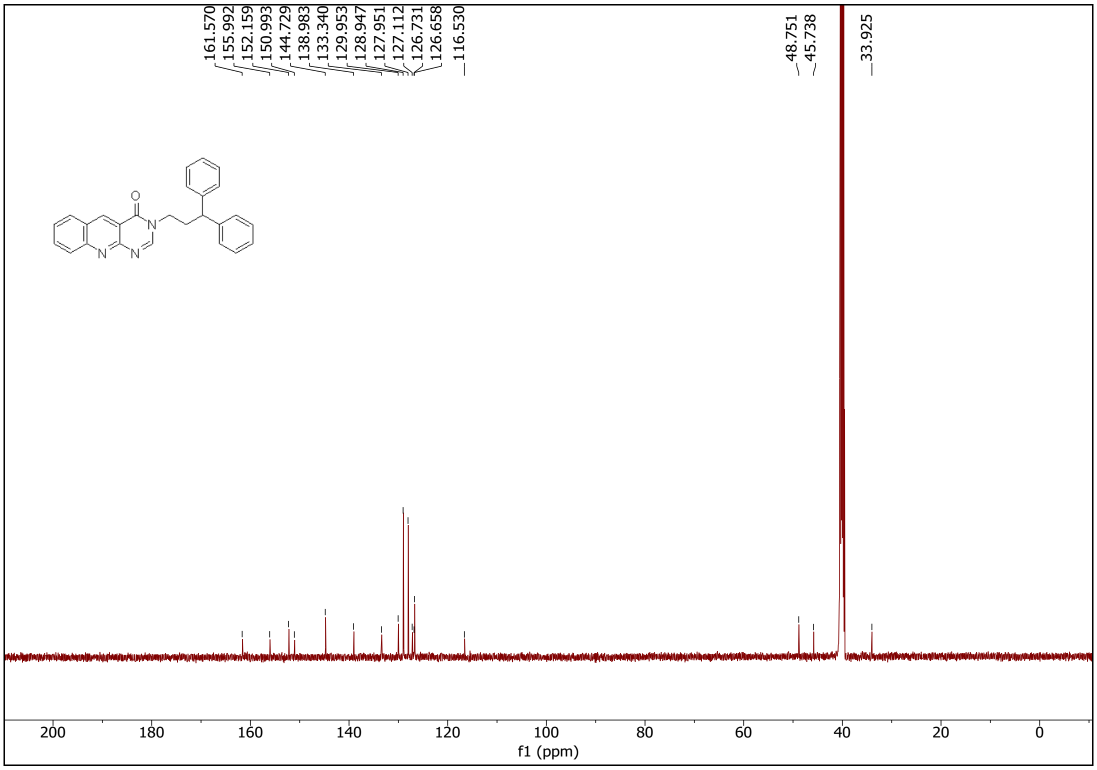

3-Cyclohexylpyrimido[4,5-*b*]quinolin-4(3*H*)-one (6e)

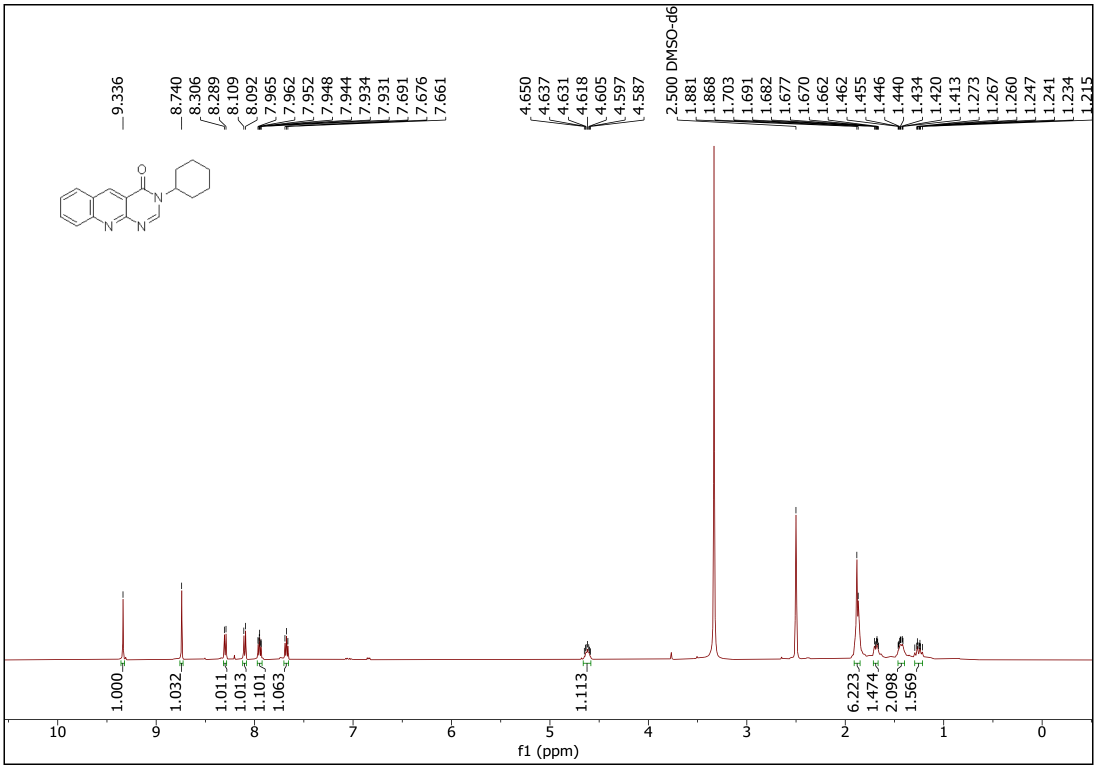

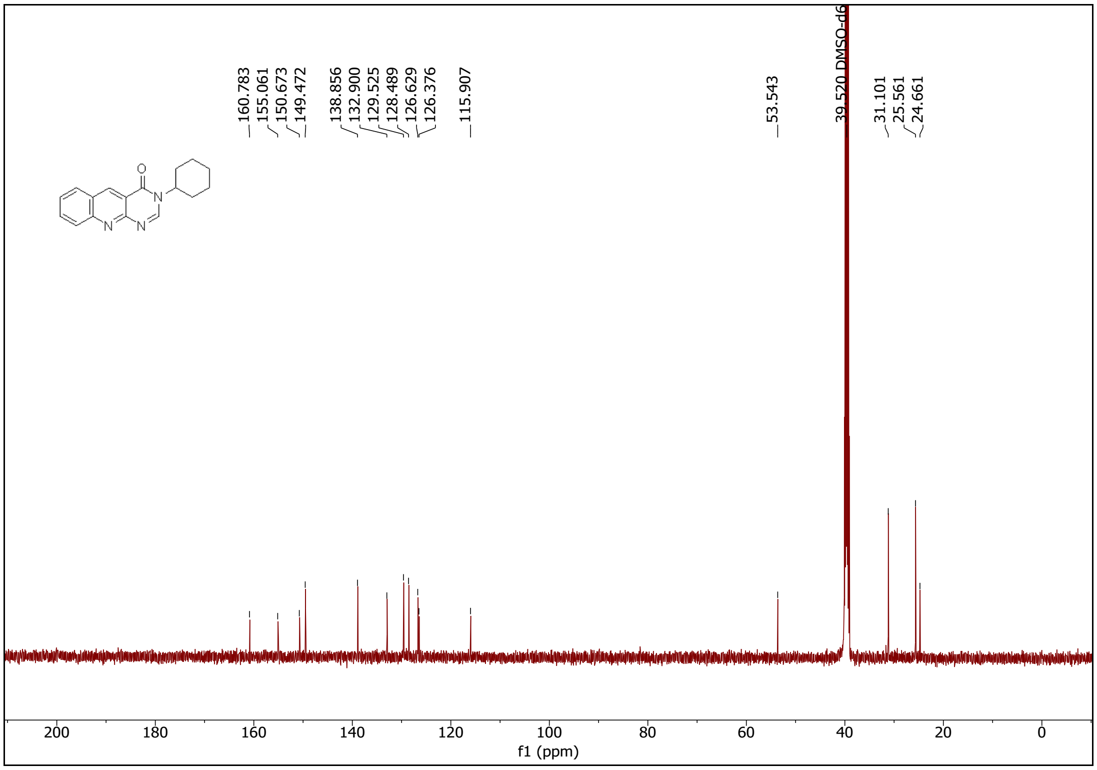

3-Benzyl-3,9-dihydro-4*H*-pyrimido[4,5-*b*]indol-4-one (7a)

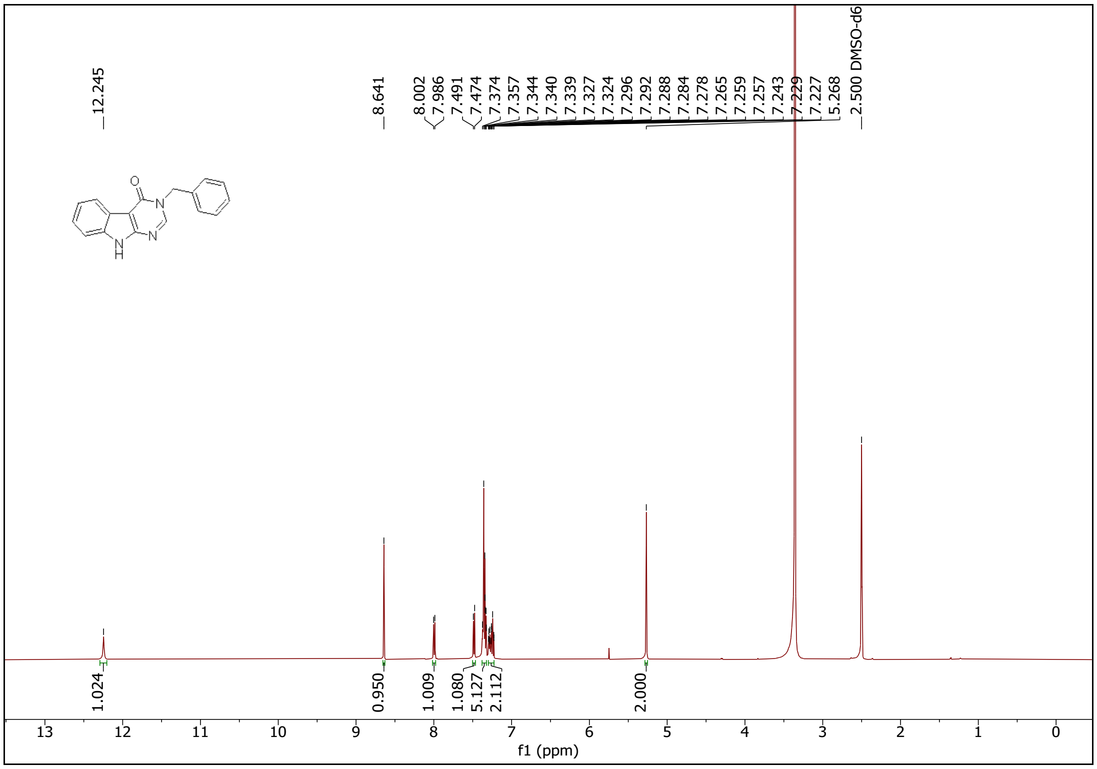

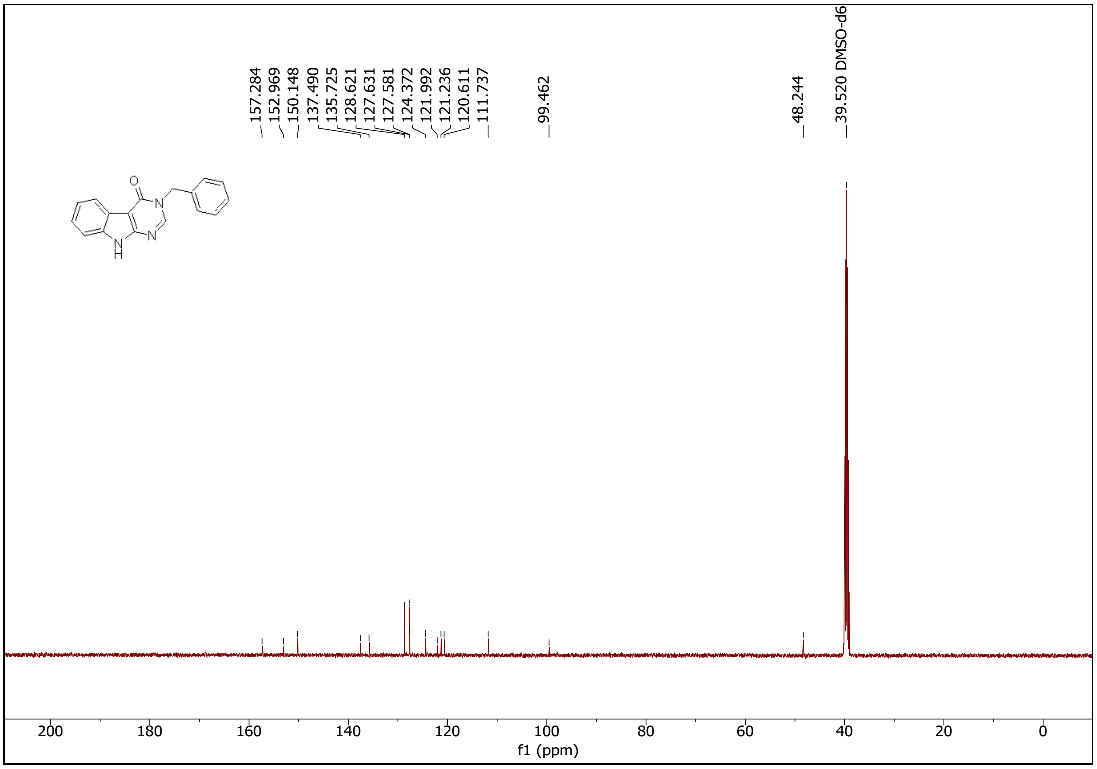

3-Butyl-3,9-dihydro-4*H*-pyrimido[4,5-*b*]indol-4-one (7b)

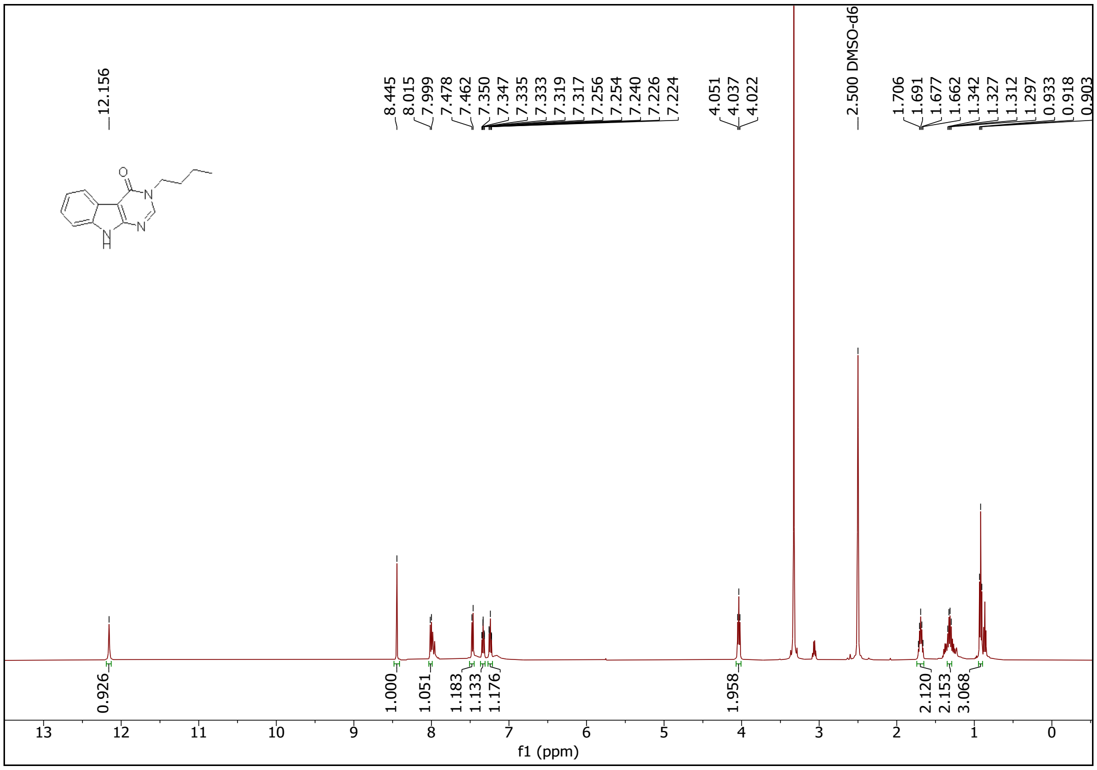

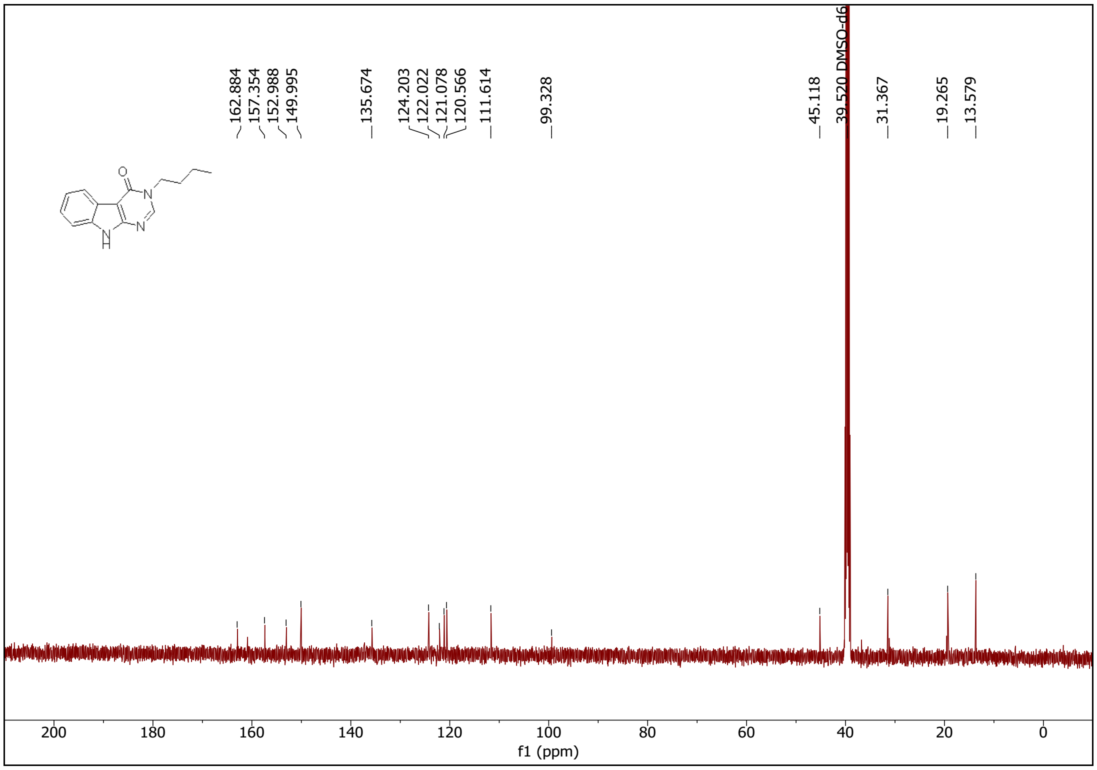

3-(2-Chlorobenzyl)-3,9-dihydro-4*H*-pyrimido[4,5-*b*]indol-4-one (7c)

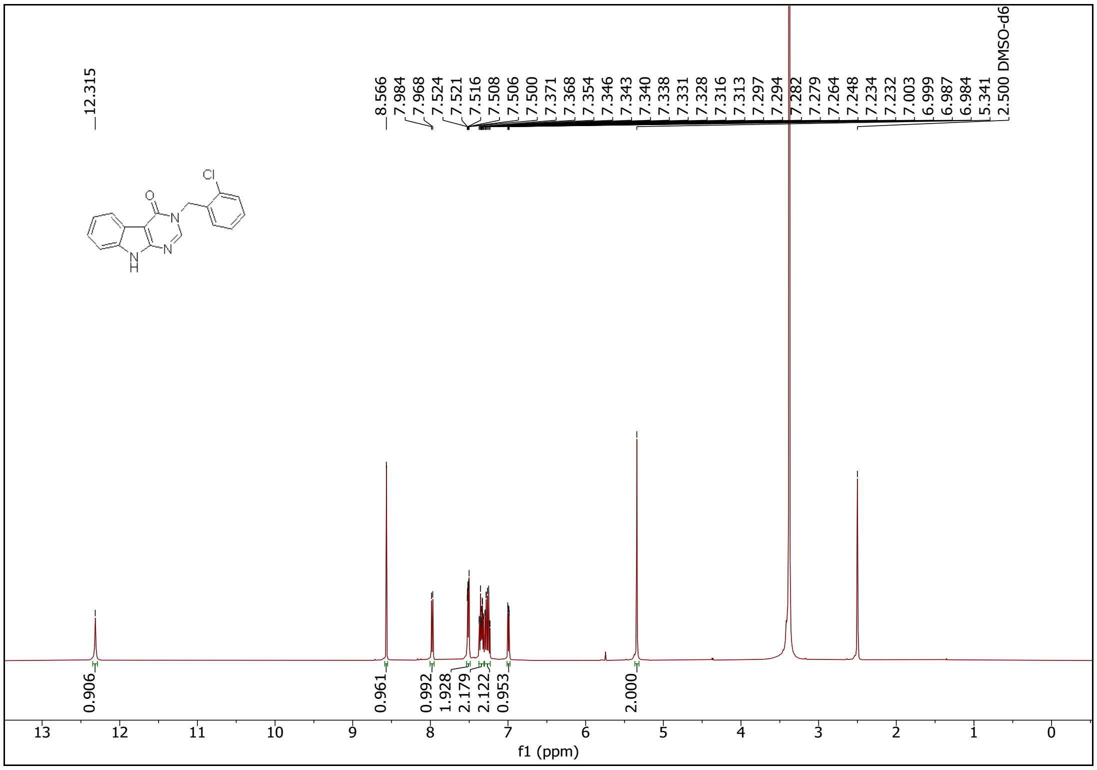

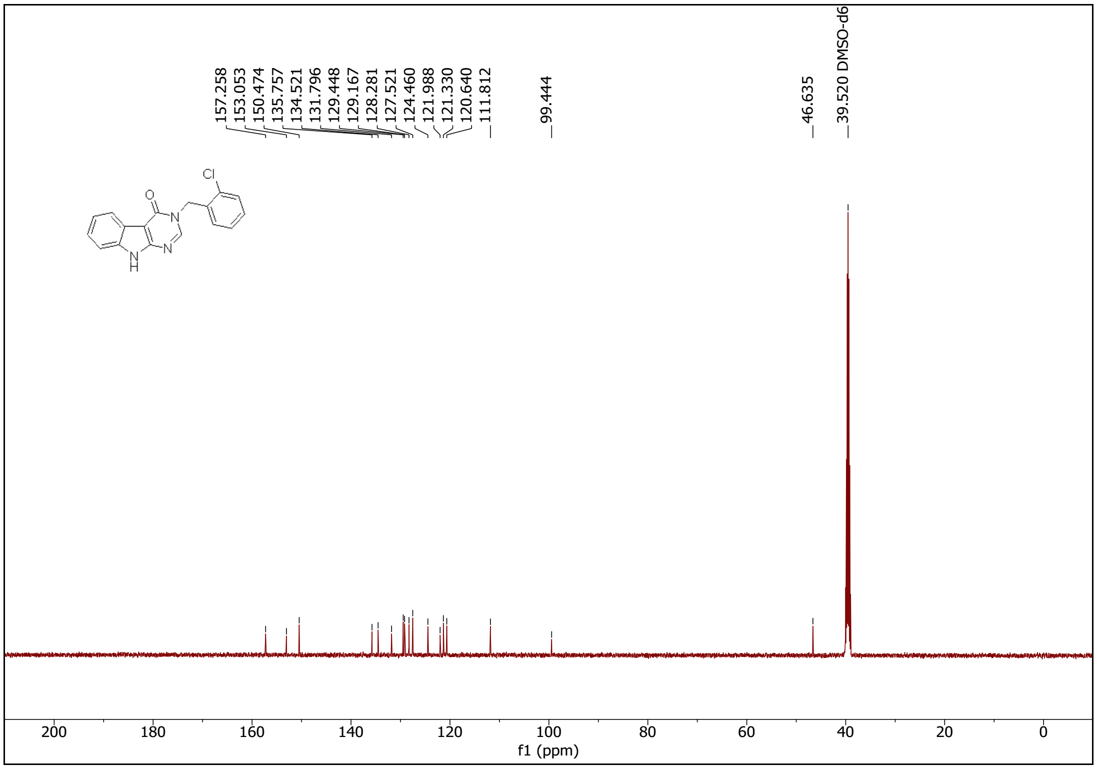

3-(4-Chlorobenzyl)-3,9-dihydro-4*H*-pyrimido[4,5-*b*]indol-4-one (7d)

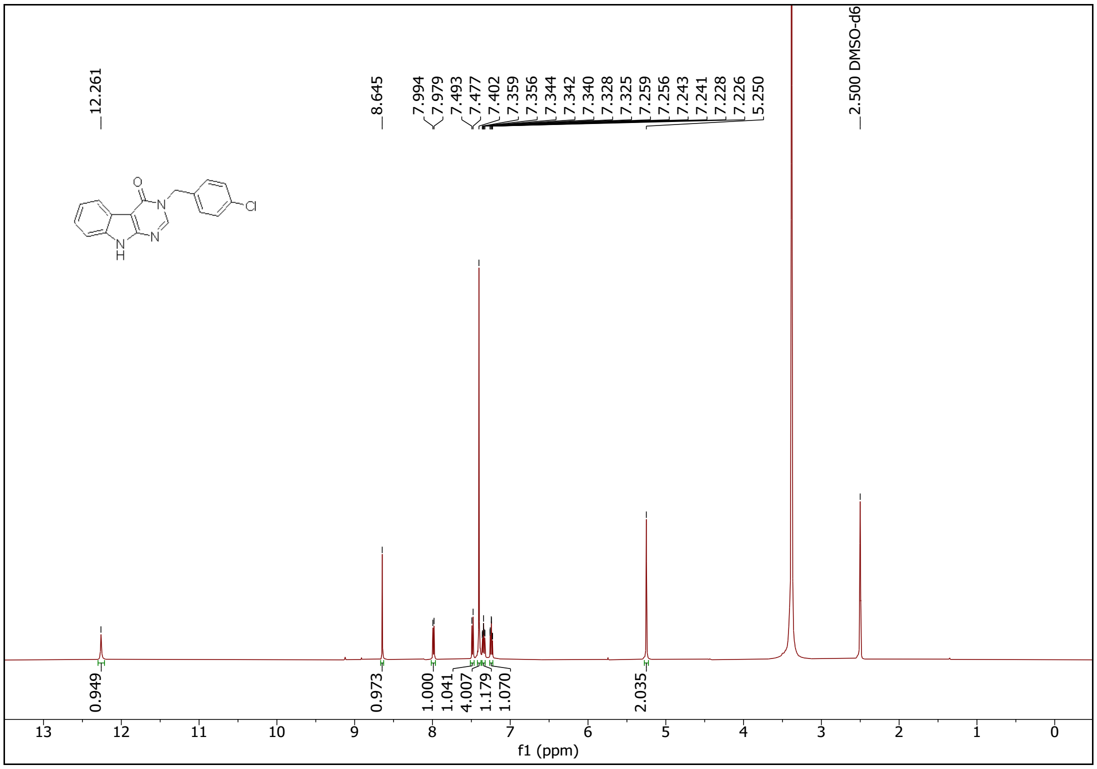

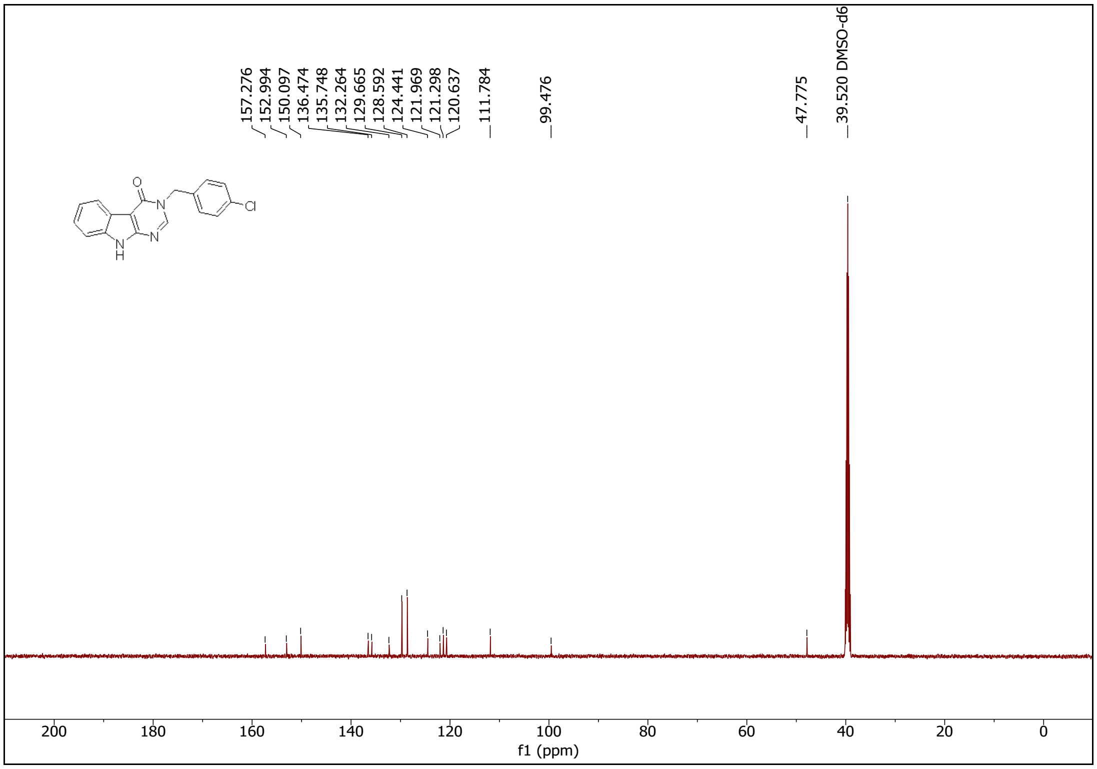

3-Cyclohexyl-3,9-dihydro-4*H*-pyrimido[4,5-*b*]indol-4-one (7e)

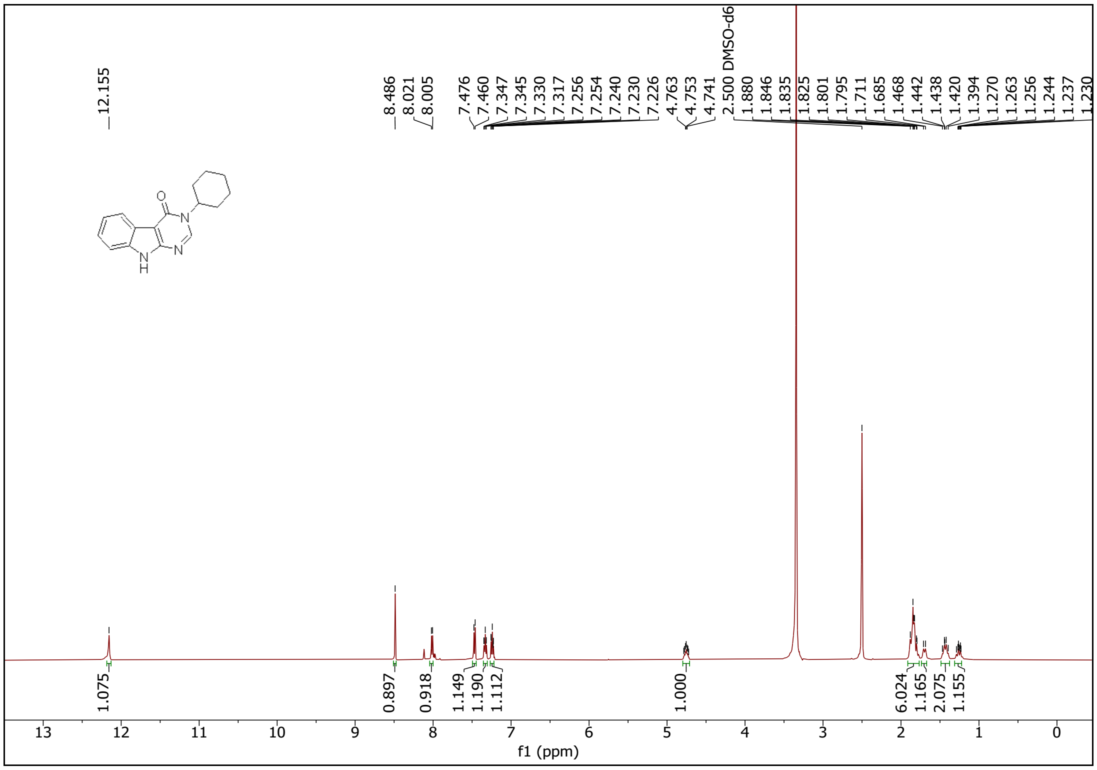

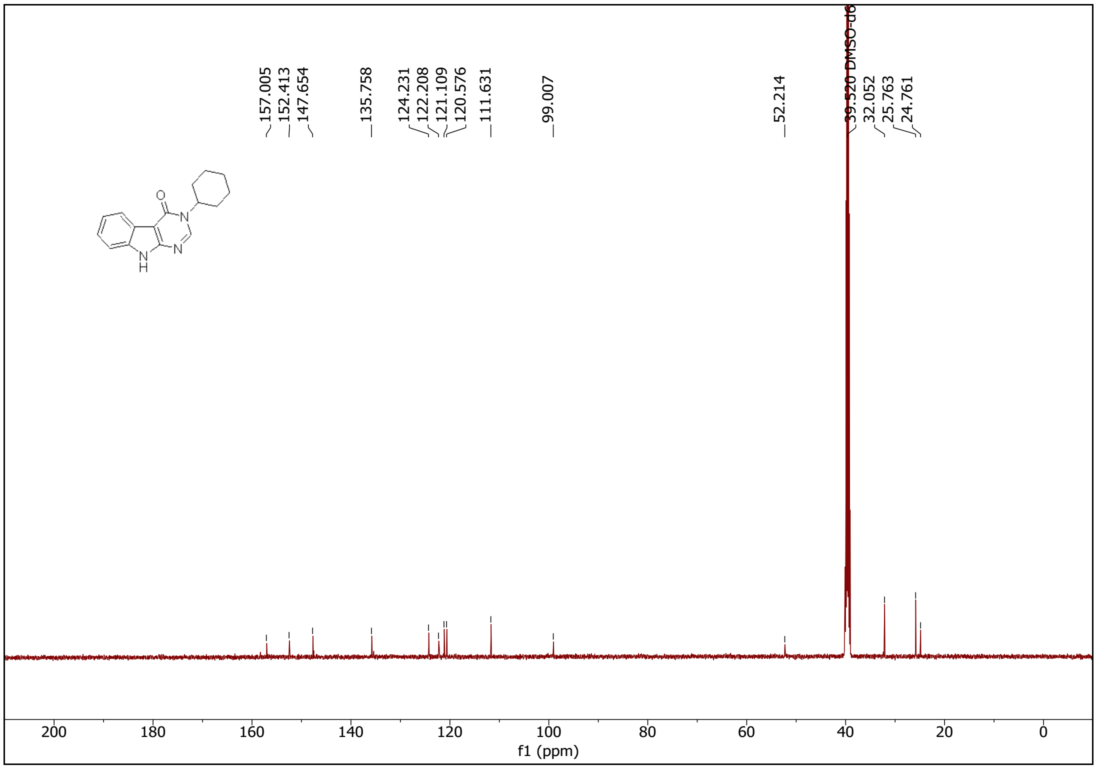

4. Representative copies of ¹H NMR spectra of the MCR-based precursors

2-Amino-*N*-butyl-5-phenylthiophene-3-carboxamide (2a)

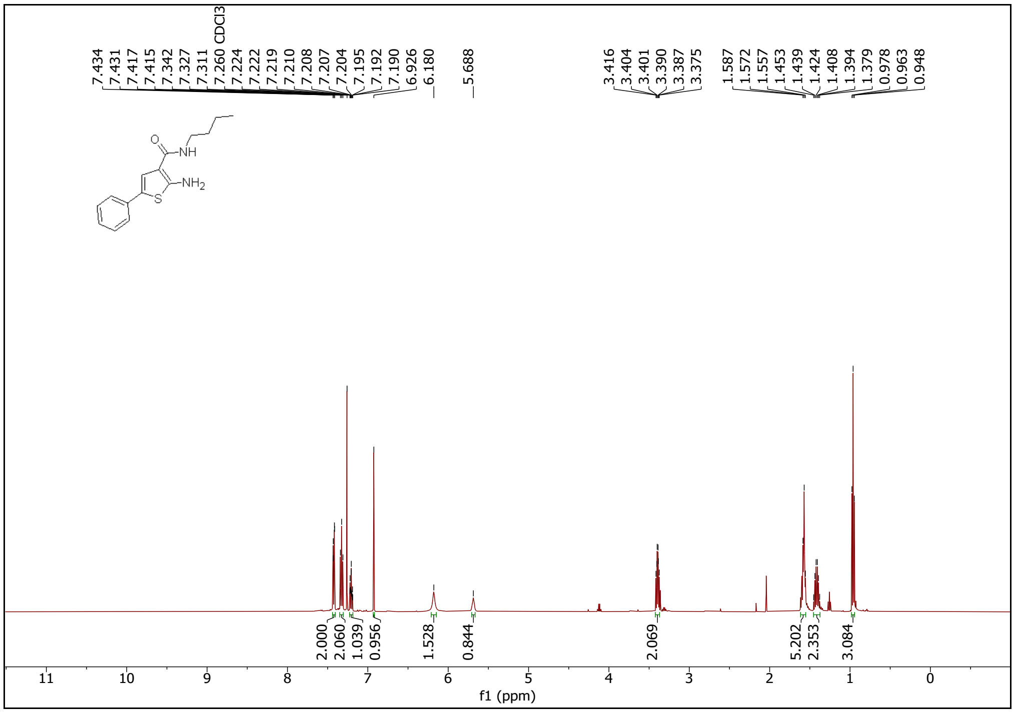

2-Amino-*N*-cyclohexyl-5-phenylthiophene-3-carboxamide (2b)

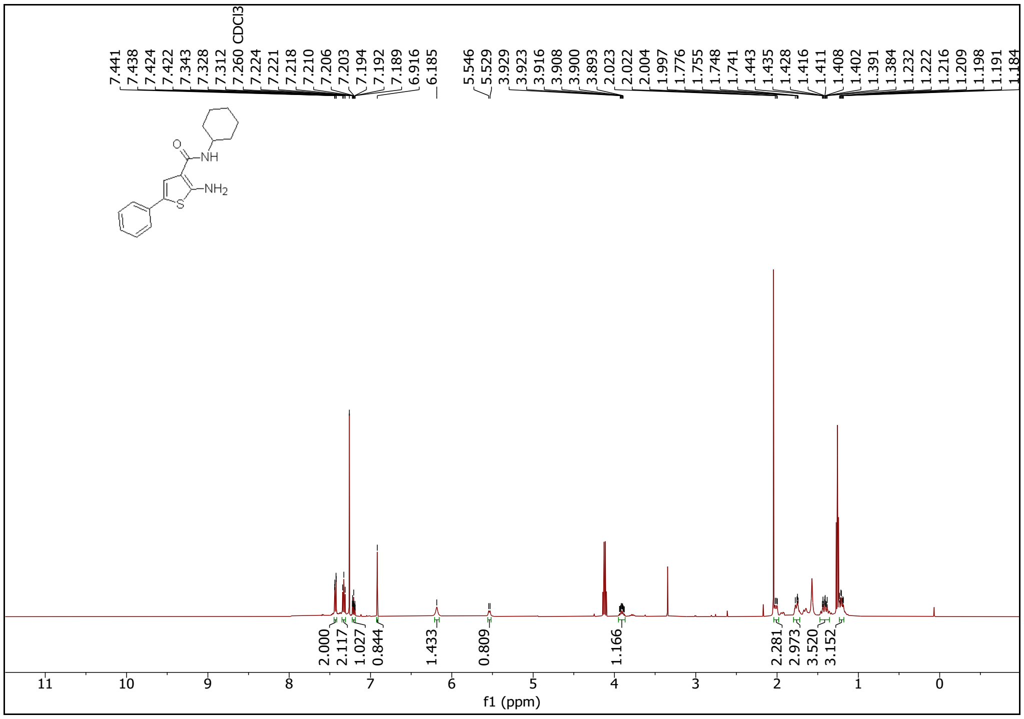

2-Amino-*N*-(naphthalen-2-ylmethyl)-5-phenylthiophene-3-carboxamide (2c)

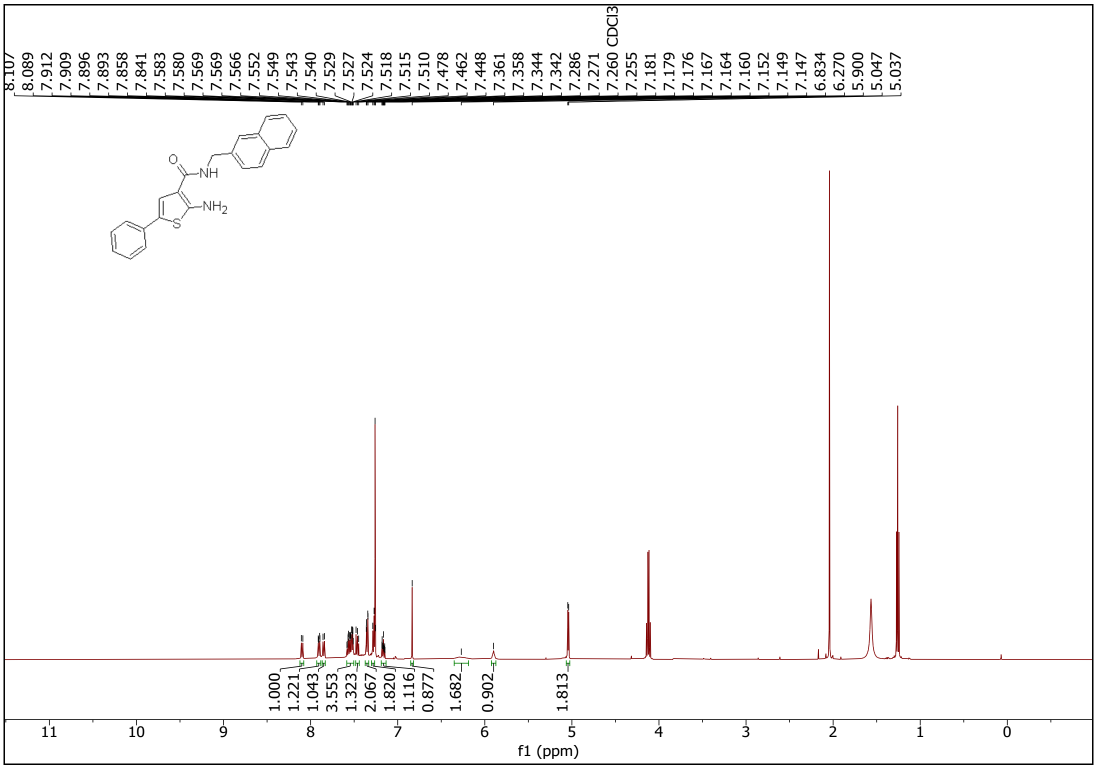

2-Amino-*N*-(furan-2-ylmethyl)-5-phenylthiophene-3-carboxamide (2d)

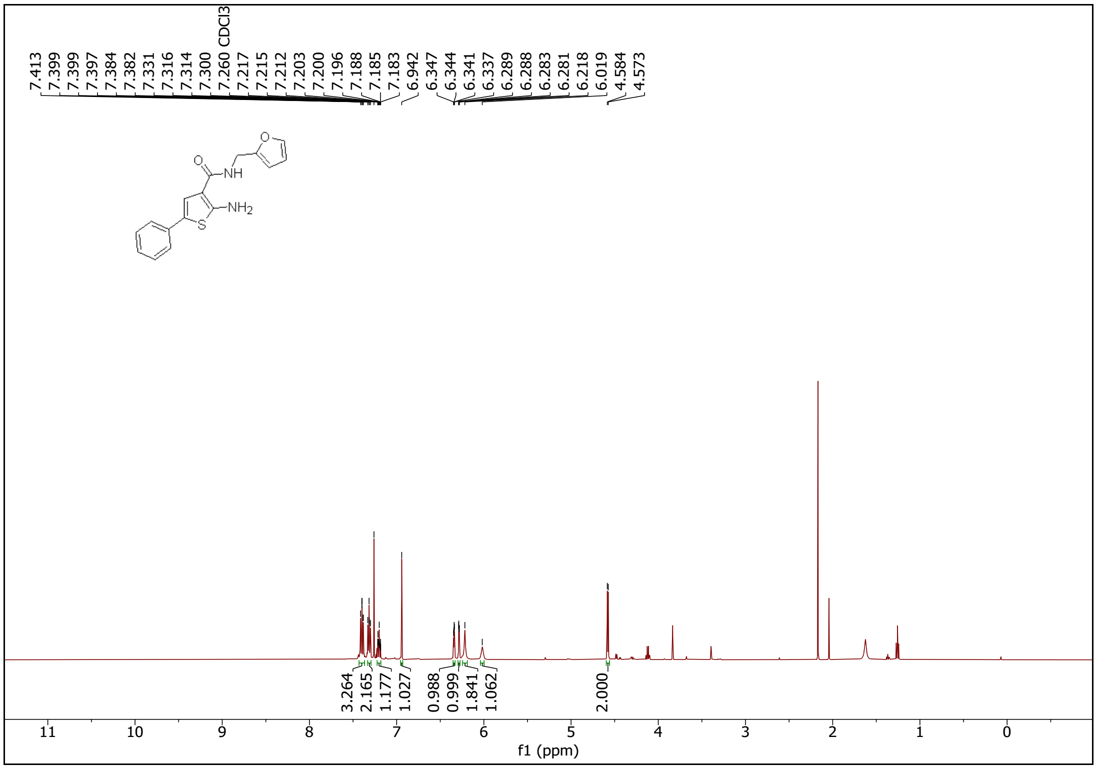

2-Amino-5-phenyl-*N*-(thiophen-2-ylmethyl)thiophene-3-carboxamide (2e)

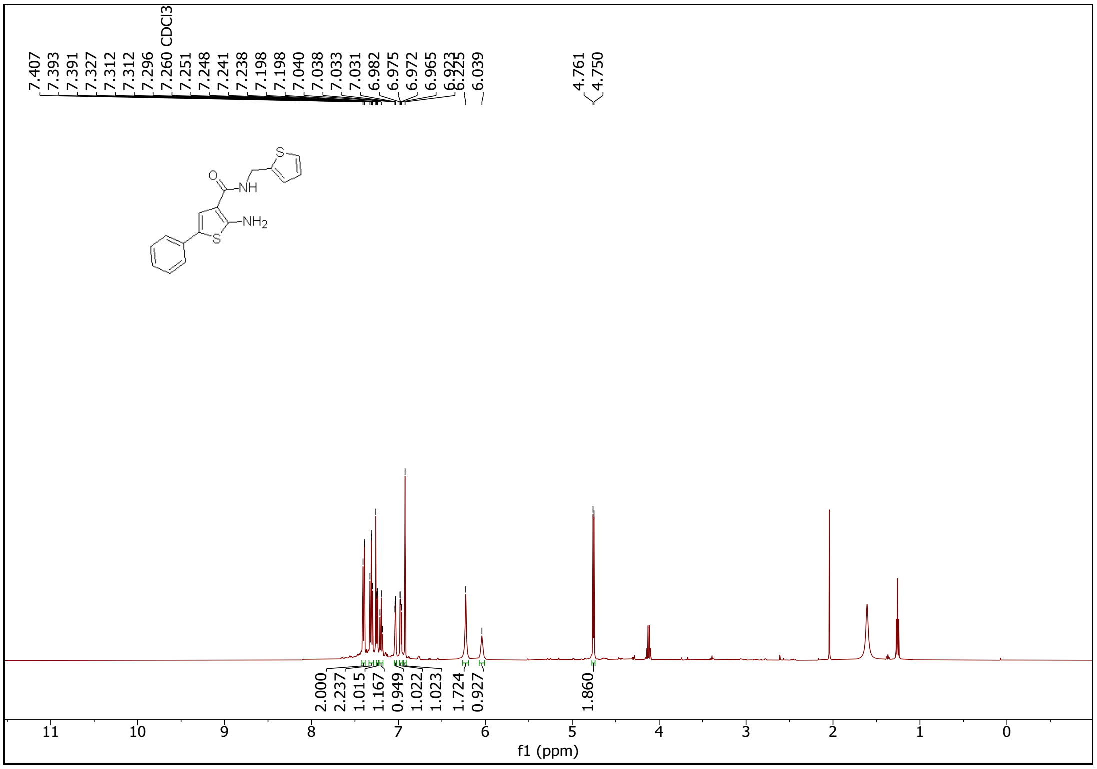

2-Amino-*N*-butylquinoline-3-carboxamide (3a)

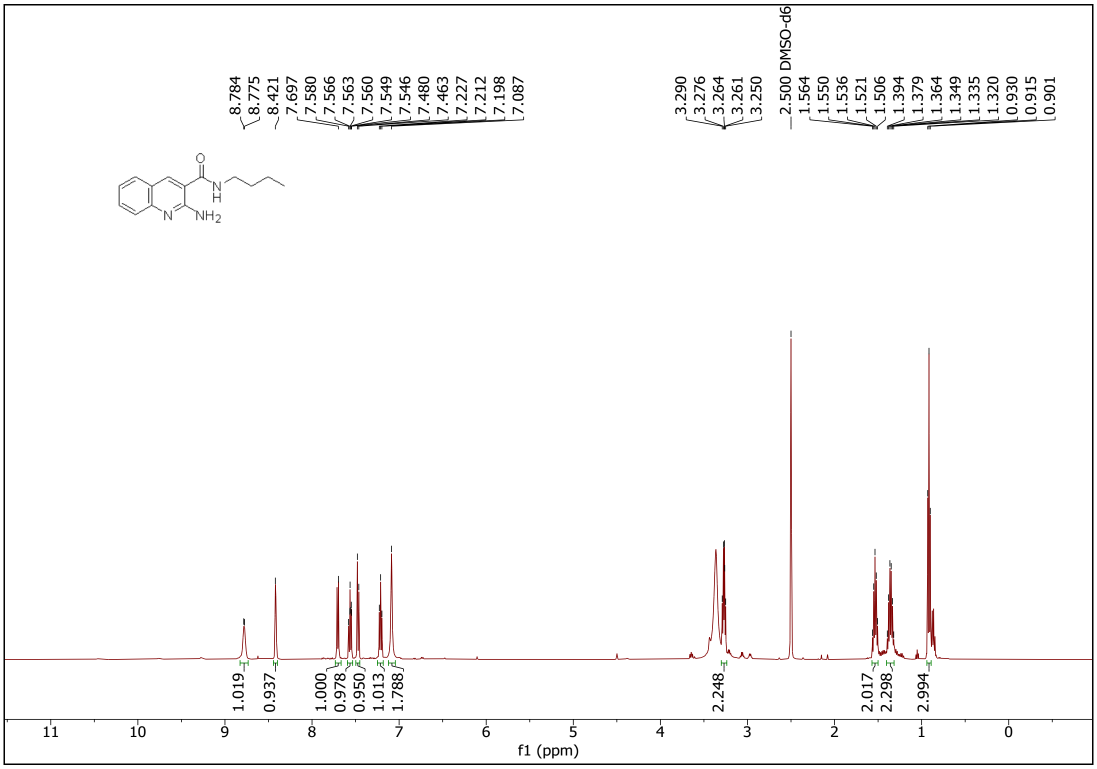

2-Amino-*N*-(furan-2-ylmethyl)quinoline-3-carboxamide (3c)

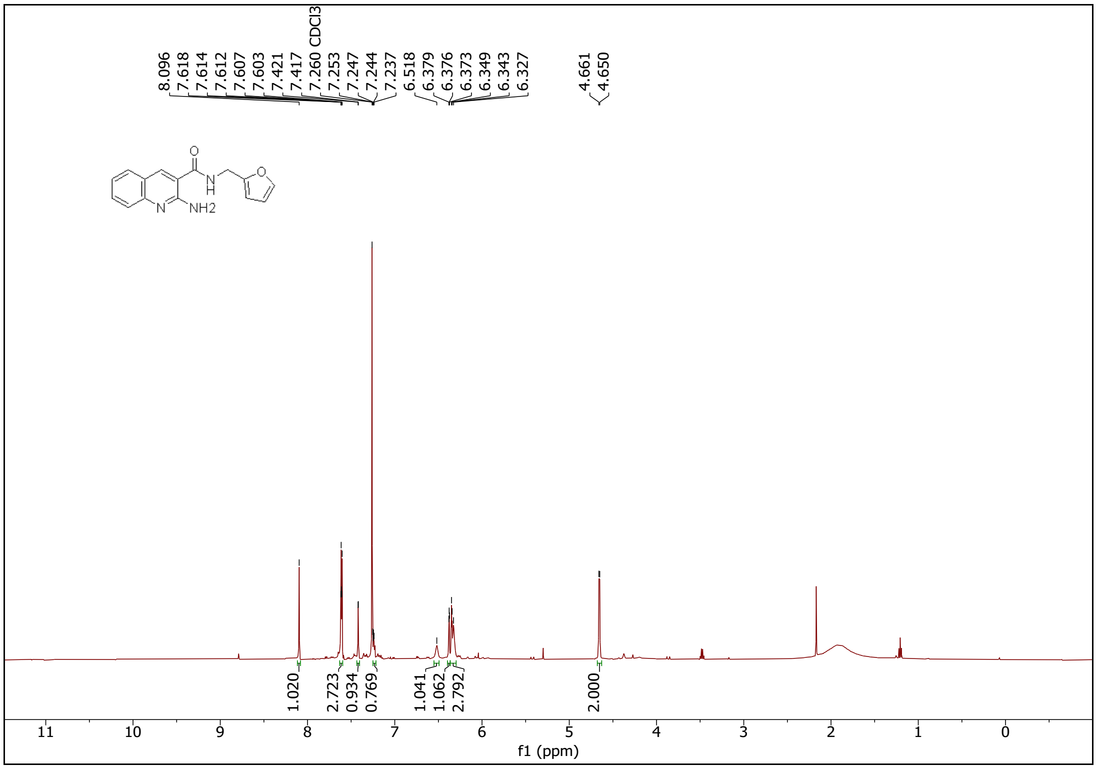

2-Amino-*N*-(3-ethoxypropyl)quinoline-3-carboxamide (3d)

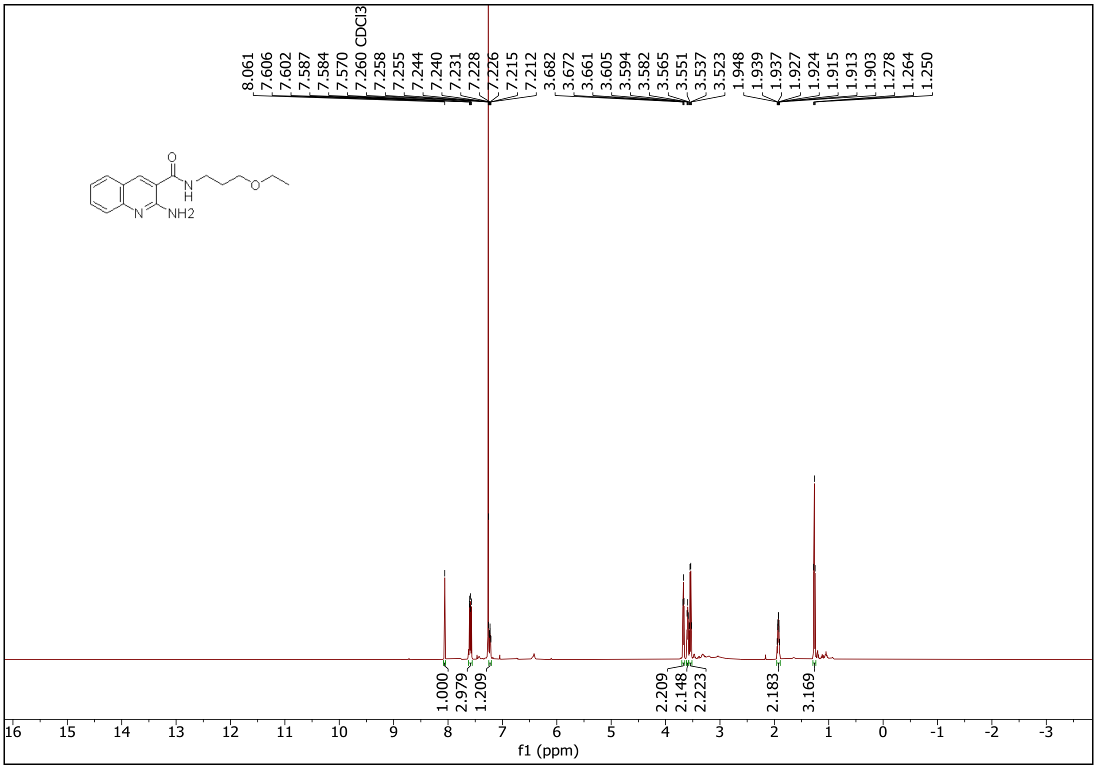

2-Amino-*N*-butyl-1*H*-indole-3-carboxamide (4a)

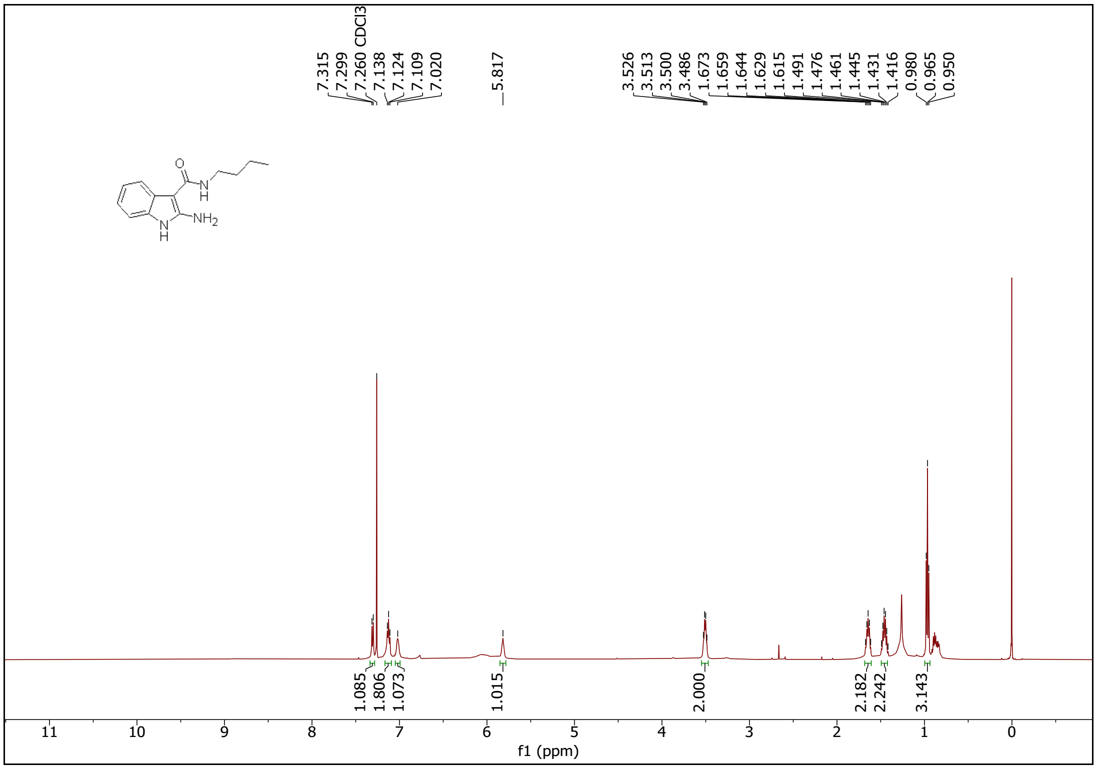

2-Amino-*N*-cyclohexyl-1*H*-indole-3-carboxamide (4b)

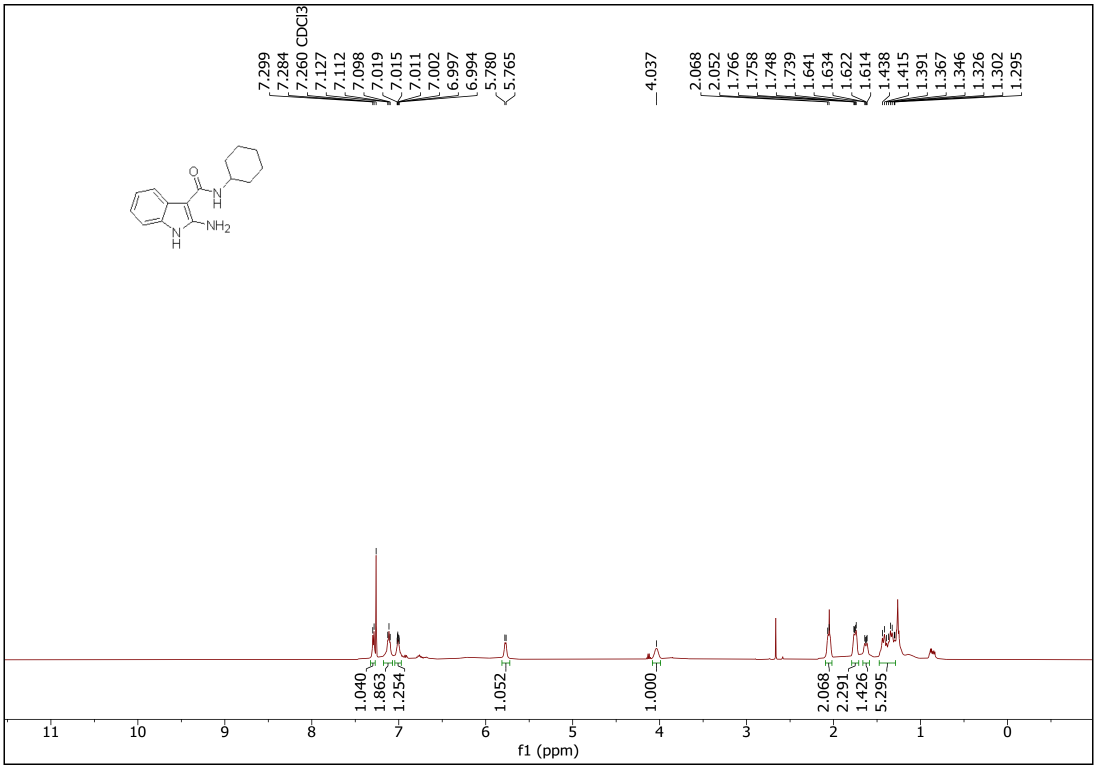

2-Amino-*N*-benzyl-1*H*-indole-3-carboxamide (4d)

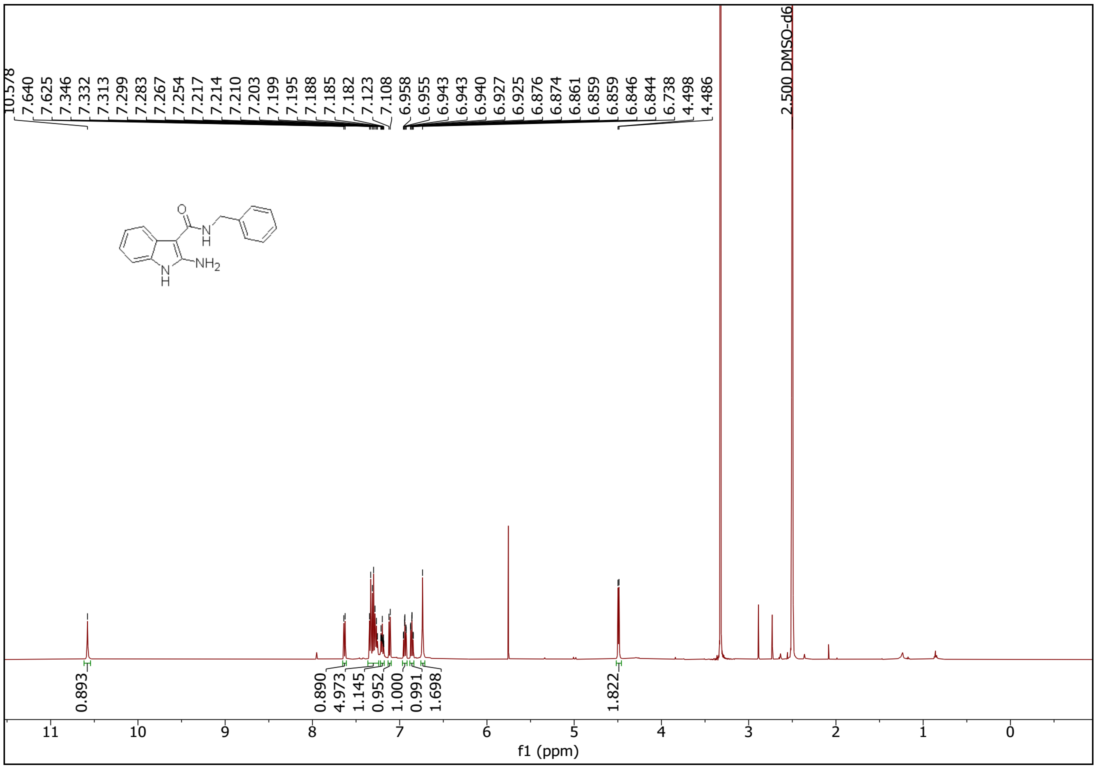

2-Amino-*N*-(4-chlorobenzyl)-1*H*-indole-3-carboxamide (4e)

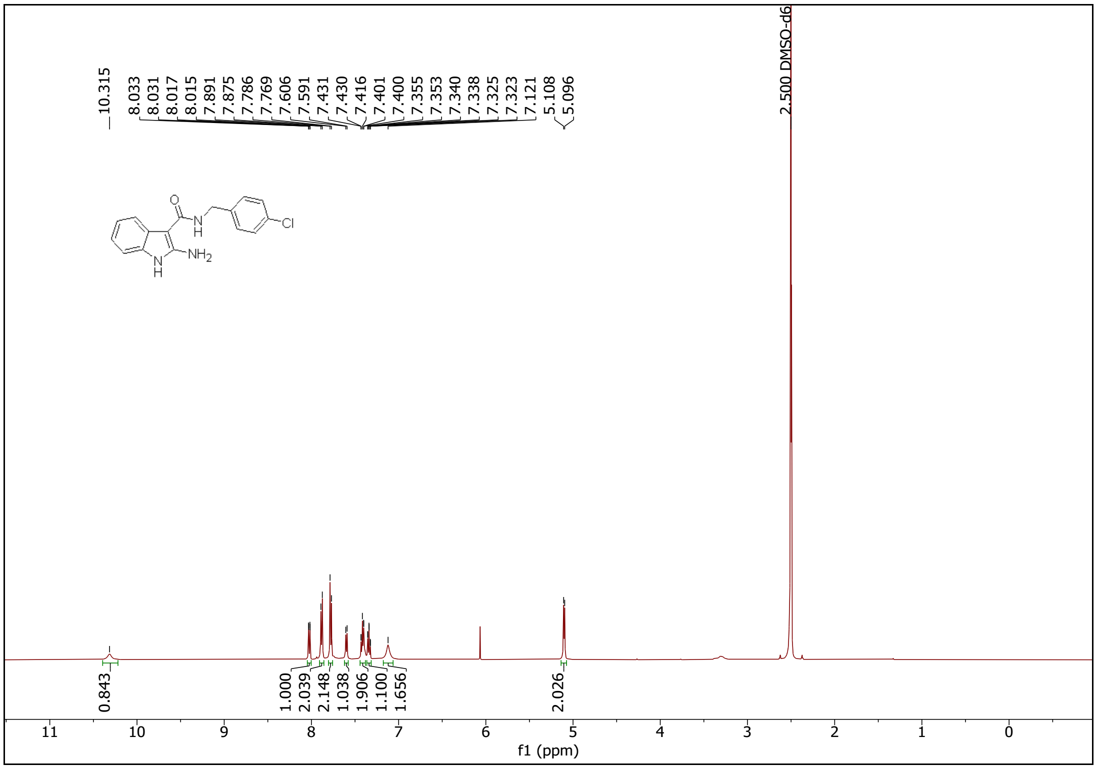

2-Amino-*N*-(2-chlorobenzyl)-1*H*-indole-3-carboxamide (4f)

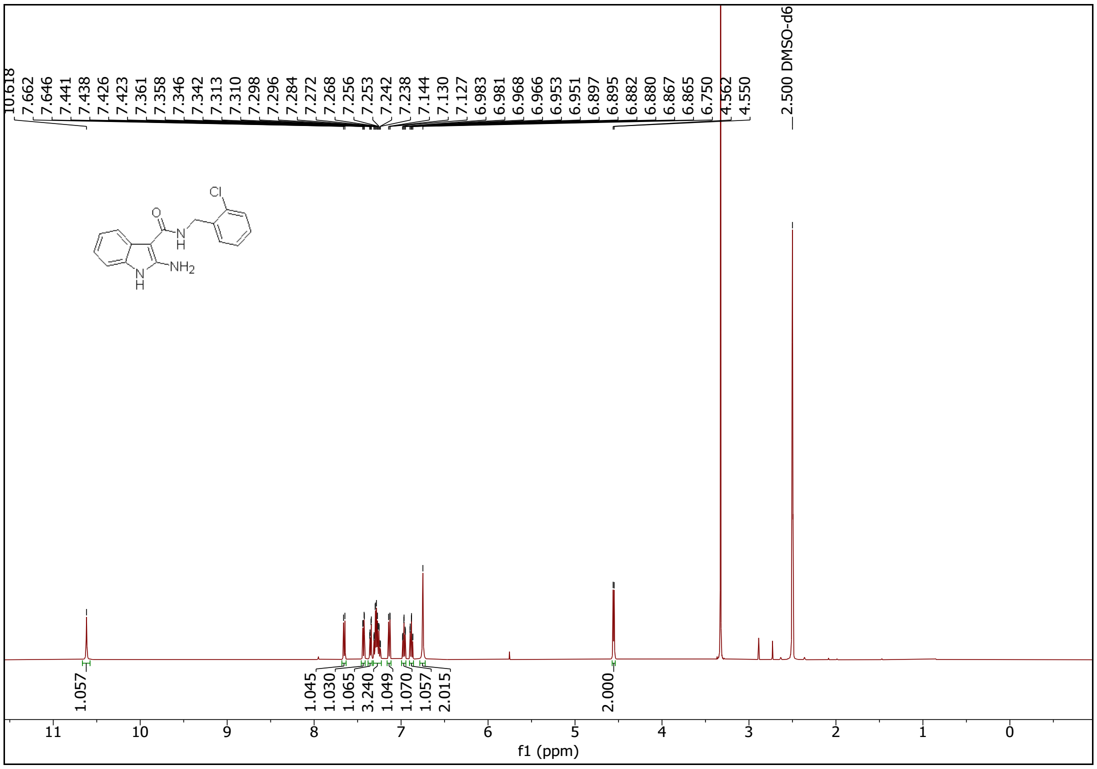

5. Procedure for absorption-fluorescence measurements

The weighed amount of each compound was dissolved in 200 μL of DMSO resulting in the production of a solution of 0.2 M. The wavelength of maximum absorption was first determined by scanning the 250–650 nm region. Then, the solution of each compound was irradiated with 330 nm (thiophenes), 284 nm (quinolones) and 310 nm (indoles) wavelength radiation and the fluorescence spectra were obtained.

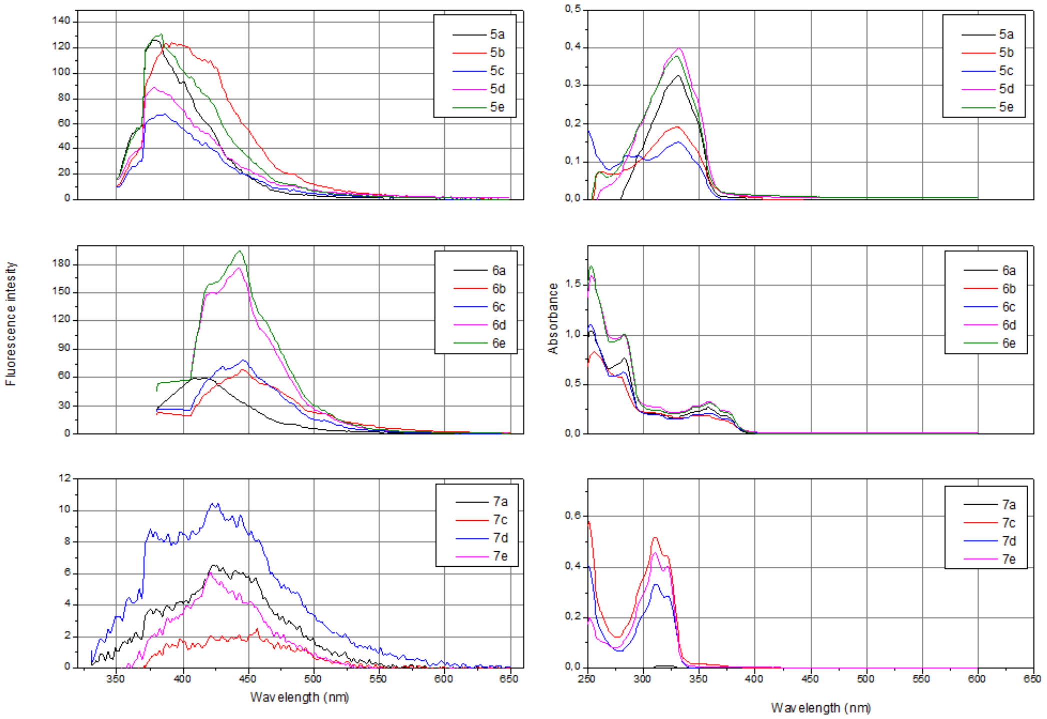

Compound	Fluorescence emission (nm)	Excitation wavelength (nm)
**5a**	377	330
**5b**	392	330
**5c**	387	330
**5d**	378	330
**5e**	384	330
**6a**	415	284
**6b**	447	284
**6c**	447	284
**6d**	442	284
**6e**	443	284
**7a**	423	310
**7c**	457	310
**7d**	427	310
**7e**	421	310

6. Single-crystal X-ray structure determination

A specimen of $C_{19}H_{21}N_3O_2$ was used for the X-ray crystallographic analysis. The X-ray intensity data were measured (λ = 1.54178 Å).

The total exposure time was 25.60 hours. The frames were integrated with the Bruker SAINT software package using a narrow-frame algorithm. The integration of the data using a monoclinic unit cell yielded a total of 14243 reflections to a maximum θ angle of 59.08° (0.90 Å resolution), of which 2688 were independent (average redundancy 5.299, completeness = 99.8%, R_{int} = 4.08%, R_{sig} = 2.81%) and 2019 (75.11%) were greater than $2\sigma(F^2)$. The final cell constants of a = 5.33600(10) Å, b = 16.4504(4) Å, c = 21.5018(6) Å, β = 94.726(2)°, volume = 1881.00(8) $Å^3$, are based upon the refinement of the XYZ-centroids of 4189 reflections above 20 σ(I) with 6.774° < 2θ < 116.5°. Data were corrected for absorption effects using the Multi-Scan method (SADABS). The ratio of minimum to maximum apparent transmission was 0.870. The calculated minimum and maximum transmission coefficients (based on crystal size) are 0.9880 and 0.9940.

The structure was solved and refined using the Bruker SHELXTL Software Package,[5] using the space group P 1 21/c 1, with Z = 4 for the formula unit, $C_{19}H_{21}N_3O_2$. The final anisotropic full-matrix least-squares refinement on F^2 with 218 variables converged at R1 = 9.68%, for the observed data and wR2 = 31.64% for all data. The goodness-of-fit was 1.085. The largest peak in the final difference electron density synthesis was 0.711 $e^-/Å^3$ and the largest hole was -0.331 $e^-/Å^3$ with an RMS deviation of 0.079 $e^-/Å^3$. On the basis of the final model, the calculated density was 1.142 g/cm^3 and F(000), 688 e^-. The CCDC access number is: 2376493.

Table S1. Sample and crystal data for **7b**.

**Identification code**	DP_24	
**Chemical formula**	$C_{19}H_{21}N_3O_2$	
**Formula weight**	323.39 g/mol	
**Temperature**	220(2) K	
**Wavelength**	1.54178 Å	
**Crystal size**	0.010 x 0.020 x 0.025 mm	
**Crystal system**	monoclinic	
**Space group**	P 1 21/c 1	
**Unit cell dimensions**	a = 5.33600(10) Å	α = 90°
	b = 16.4504(4) Å	β = 94.726(2)°
	c = 21.5018(6) Å	γ = 90°
**Volume**	1881.00(8) $Å^3$	
**Z**	4	
**Density (calculated)**	1.142 g/cm^3	
**Absorption coefficient**	0.606 mm^{-1}	
**F(000)**	688	

Table 2. Data collection and structure refinement for **7b**.

**Theta range for data collection**	3.39 to 59.08°	
**Index ranges**	-5<=h<=5, -18<=k<=16, -22<=l<=23	
**Reflections collected**	14243	
**Independent reflections**	2688 [R(int) = 0.0408]	
**Coverage of independent reflections**	99.8%	
**Absorption correction**	Multi-Scan	
**Max. and min. transmission**	0.9940 and 0.9880	
**Refinement method**	Full-matrix least-squares on F^2	
**Function minimized**	Σ w(F_o^2 - F_c^2)^2	
**Data / restraints / parameters**	2688 / 0 / 218	
**Goodness-of-fit on F^2**	1.085	
**Final R indices**	2019 data; I>2σ(I)	R1 = 0.0968, wR2 = 0.2876
	all data	R1 = 0.1198, wR2 = 0.3164
**Weighting scheme**	w=1/[σ^2(F_o^2)+(0.1848P)^2+2.2846P] where P=(F_o^2+2F_c^2)/3	
**Largest diff. peak and hole**	0.711 and -0.331 eÅ^{-3}	
**R.M.S. deviation from mean**	0.079 eÅ^{-3}	

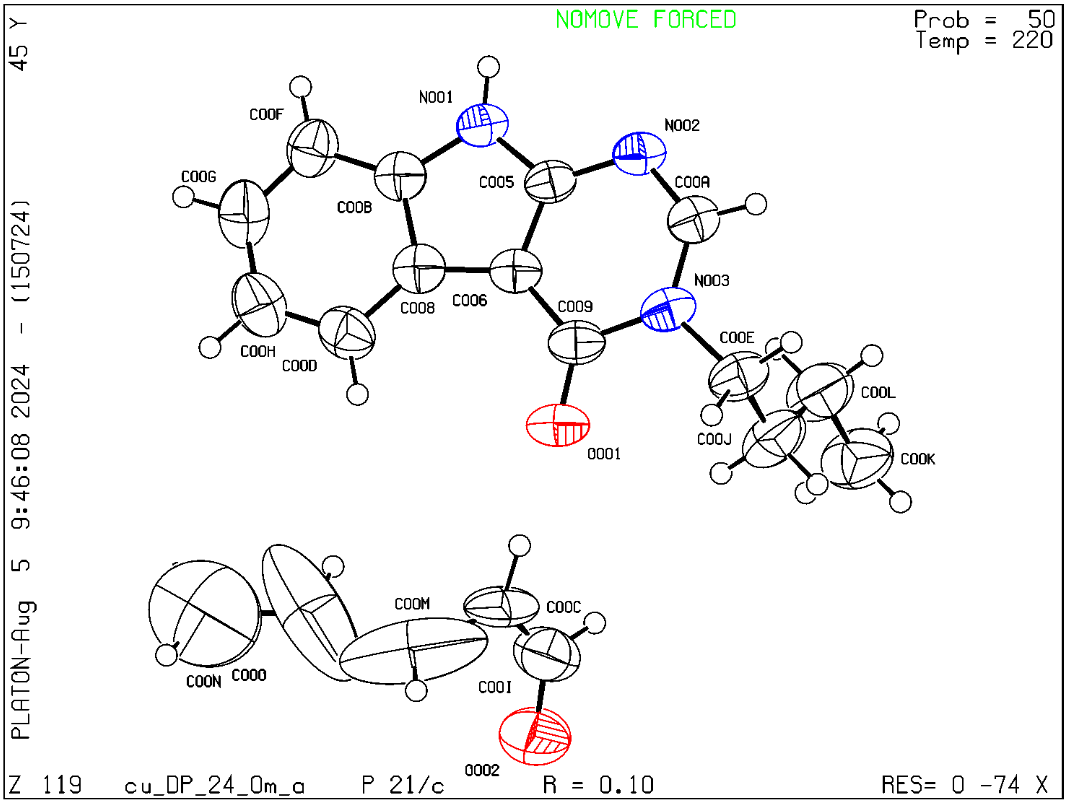

References

(1) Wang, K.; Nguyen, K.; Huang, Y.; Dömling, A. Cyanoacetamide Multicomponent Reaction (I): Parallel Synthesis of Cyanoacetamides. J Comb Chem 2009, 11 (5), 920–927. https://doi.org/10.1021/cc9000778.

(2) Wang, K.; Kim, D.; Dömling, A. Cyanoacetamide MCR (III): Three-Component Gewald Reactions Revisited. J Comb Chem 2010, 12 (1), 111–118. https://doi.org/10.1021/cc9001586.

(3) Wang, K.; Herdtweck, Eberhardt, and Dömling, A. Cyanoacetamides (IV): Versatile One-Pot Route to 2-Quinoline-3- Carboxamides. 2012, 14 (Iv), 316–322.

(4) Wang, K.; Herdtweck, E.; Dömling, A. One-Pot Synthesis of 2-Amino-Indole-3-Carboxamide and Analogous. ACS Comb Sci 2011, 13 (2), 140–146. https://doi.org/10.1021/co100040z.

(5) Sheldrick, G. M. Crystal Structure Refinement with SHELXL. Acta Crystallogr C Struct Chem 2015, 71 (1), 3–8. https://doi.org/10.1107/S2053229614024218.
