# Supplementary material for: Heteroannulations of cyanoacetamide-based MCR scaffolds utilizing formamide
Source: Beilstein J Org Chem. 2025 Jan 24;21:217–25. doi: 10.3762/bjoc.21.13 (PMC11773184; doi:10.3762/bjoc.21.13)

BEILSTEIN JOURNAL OF ORGANIC CHEMISTRY

Supporting Information

for

Heteroannulations of cyanoacetamide-based MCR scaffolds utilizing formamide

Marios Zingiridis, Danae Papachristodoulou, Despoina Menegaki,
Konstantinos G. Froudas and Constantinos G. Neochoritis

Beilstein J. Org. Chem. **2025,** *21,* 217–225. doi:10.3762/bjoc.21.13

CheckCIF report for 7b

License and Terms: This is a supporting information file under the terms of the Creative Commons Attribution License (https://creativecommons.org/licenses/by/4.0). Please note that the reuse, redistribution and reproduction in particular requires that the author(s) and source are credited and that individual graphics may be subject to special legal provisions.
The license is subject to the *Beilstein Journal of Organic Chemistry* terms and conditions: (https://www.beilstein-journals.org/bjoc/terms)

checkCIF/PLATON report

You have not supplied any structure factors. As a result the full set of tests cannot be run.

THIS REPORT IS FOR GUIDANCE ONLY. IF USED AS PART OF A REVIEW PROCEDURE FOR PUBLICATION, IT SHOULD NOT REPLACE THE EXPERTISE OF AN EXPERIENCED CRYSTALLOGRAPHIC REFEREE.

No syntax errors found. CIF dictionary Interpreting this report

Datablock: cu_DP_24_0m_a

Bond precision:	C-C = 0.0095 A		Wavelength=1.54178
Cell:	a=5.3360(1)	b=16.4504(4)	c=21.5018(6)
	alpha=90	beta=94.726(2)	gamma=90
Temperature:	220 K		

	Calculated	Reported
Volume	1881.00(8)	1881.00(8)
Space group	P 21/c	P 21/c
Hall group	-P 2ybc	-P 2ybc
Moiety formula	C14 H15 N3 O, C3 H3 O, C2 H3	C14 H15 N3 O, C5 H6 O
Sum formula	C19 H21 N3 O2	C19 H21 N3 O2
Mr	323.39	323.39
Dx,g cm-3	1.142	1.142
Z	4	4
Mu (mm-1)	0.606	0.606
F000	688.0	688.0
F000'	690.01	
h,k,lmax	5,18,23	5,18,23
Nref	2694	2688
Tmin,Tmax	0.986,0.994	0.654,0.752
Tmin'	0.985	

Correction method= # Reported T Limits: Tmin=0.654 Tmax=0.752
AbsCorr = MULTI-SCAN

Data completeness= 0.998 Theta(max)= 59.081

R(reflections)= 0.0968(2019) wR2(reflections)= 0.3164(2688)

S = 1.085 Npar= 218

The following ALERTS were generated. Each ALERT has the format
test-name_ALERT_alert-type_alert-level.
Click on the hyperlinks for more details of the test.

Alert level B

```
THETM01_ALERT_3_B  The value of sine(theta_max)/wavelength is less than 0.575
            Calculated sin(theta_max)/wavelength =    0.5564
```

Author Response: These were weakly diffracting crystals, especially in higher angles. Although a high-brilliance IuS microfocus with Cu radiation was used reasonable diffraction data could be obtained only to moderate resolution, despite multiple attempts to improve crystal quality.

Alert level C

```
DIFMX02_ALERT_1_C  The maximum difference density is > 0.1*ZMAX*0.75
            The relevant atom site should be identified.
PLAT042_ALERT_1_C Calc. and Reported MoietyFormula Strings  Differ     Please Check
              Calc: C14 H15 N3 O, C3 H3 O, C2 H3
              Rep.: C14 H15 N3 O, C5 H6 O
PLAT084_ALERT_3_C High wR2 Value (i.e. > 0.25) ..................       0.32 Report
PLAT094_ALERT_2_C Ratio of Maximum / Minimum Residual Density ....       2.15 Report
PLAT097_ALERT_2_C Large Reported Max.  (Positive) Residual Density       0.71 eA-3
PLAT230_ALERT_2_C Hirshfeld Test Diff for    C00E     --C00J     .        5.4 s.u.
PLAT234_ALERT_4_C Large Hirshfeld Difference C00K     --C00L     .       0.16 Ang.
PLAT244_ALERT_4_C Low    'Solvent' Ueq as Compared to Neighbors of       C00C Check
PLAT260_ALERT_2_C Large Average Ueq of Residue Including      O002      0.122 Check
PLAT260_ALERT_2_C Large Average Ueq of Residue Including      C00N      0.252 Check
PLAT340_ALERT_3_C Low Bond Precision on  C-C Bonds ..............    0.00947 Ang.
```

Alert level G

```
PLAT007_ALERT_5_G Number of Unrefined Donor-H Atoms .............          1 Report
              H001
PLAT012_ALERT_1_G N.O.K.   _shelx_res_checksum Found in CIF ......     Please Check
PLAT344_ALERT_2_G Unusual sp?       Angle Range in Solvent/Ion for       C00M Check
PLAT344_ALERT_2_G Unusual sp?       Angle Range in Solvent/Ion for       C00O Check
PLAT367_ALERT_2_G Long?  C(sp?)-C(sp?) Bond  C00C     - C00M     .       1.60 Ang.
PLAT367_ALERT_2_G Long?  C(sp?)-C(sp?) Bond  C00N     - C00O     .       1.55 Ang.
PLAT432_ALERT_2_G Short Inter X...Y Contact  O001     ..C00C     .       2.86 Ang.
                                                  x,y,z  =      1_555 Check
PLAT432_ALERT_2_G Short Inter X...Y Contact  C00C     ..C00O     .       2.74 Ang.
                                                  x,y,z  =      1_555 Check
PLAT432_ALERT_2_G Short Inter X...Y Contact  C00M     ..C00O     .       1.77 Ang.
                                                  x,y,z  =      1_555 Check
PLAT720_ALERT_4_G Number of Unusual/Non-Standard Labels ..........         45 Note
              O001     O002     N001     H001     N002     N003     C00A     H00A
              C00B     C00C     H00C     C00D     H00D     C00E     H00B     H00E
              C00F     H00F     C00G     H00G     C00H     H00H     C00I     H00I
              C00J     H00J     H00K     C00K     H00L     H00M     H00N     C00L
              H00O     H00P     C00M     H00Q     C00N     H00R     H00S     C00O
```

```
             H00T    C005    C006    C008    C009
PLAT773_ALERT_2_G Check long C-C Bond in CIF: C00M     --C00O           1.76 Ang.
PLAT790_ALERT_4_G Centre of Gravity not Within Unit Cell: Resd.  #         3 Note
             C2 H3
PLAT883_ALERT_1_G No Info/Value for _atom_sites_solution_primary .     Please Do !
```

```
   0 ALERT level A = Most likely a serious problem - resolve or explain
   1 ALERT level B = A potentially serious problem, consider carefully
  11 ALERT level C = Check. Ensure it is not caused by an omission or oversight
  13 ALERT level G = General information/check it is not something unexpected

   4 ALERT type 1 CIF construction/syntax error, inconsistent or missing data
  13 ALERT type 2 Indicator that the structure model may be wrong or deficient
   3 ALERT type 3 Indicator that the structure quality may be low
   4 ALERT type 4 Improvement, methodology, query or suggestion
   1 ALERT type 5 Informative message, check
```

It is advisable to attempt to resolve as many as possible of the alerts in all categories. Often the minor alerts point to easily fixed oversights, errors and omissions in your CIF or refinement strategy, so attention to these fine details can be worthwhile. In order to resolve some of the more serious problems it may be necessary to carry out additional measurements or structure refinements. However, the purpose of your study may justify the reported deviations and the more serious of these should normally be commented upon in the discussion or experimental section of a paper or in the "special_details" fields of the CIF. checkCIF was carefully designed to identify outliers and unusual parameters, but every test has its limitations and alerts that are not important in a particular case may appear. Conversely, the absence of alerts does not guarantee there are no aspects of the results needing attention. It is up to the individual to critically assess their own results and, if necessary, seek expert advice.

Publication of your CIF in IUCr journals

A basic structural check has been run on your CIF. These basic checks will be run on all CIFs submitted for publication in IUCr journals (*Acta Crystallographica*, *Journal of Applied Crystallography*, *Journal of Synchrotron Radiation*); however, if you intend to submit to *Acta Crystallographica Section C* or *E* or *IUCrData*, you should make sure that full publication checks are run on the final version of your CIF prior to submission.

Publication of your CIF in other journals

Please refer to the *Notes for Authors* of the relevant journal for any special instructions relating to CIF submission.

PLATON version of 15/07/2024; check.def file version of 15/07/2024

Datablock cu_DP_24_0m_a - ellipsoid plot

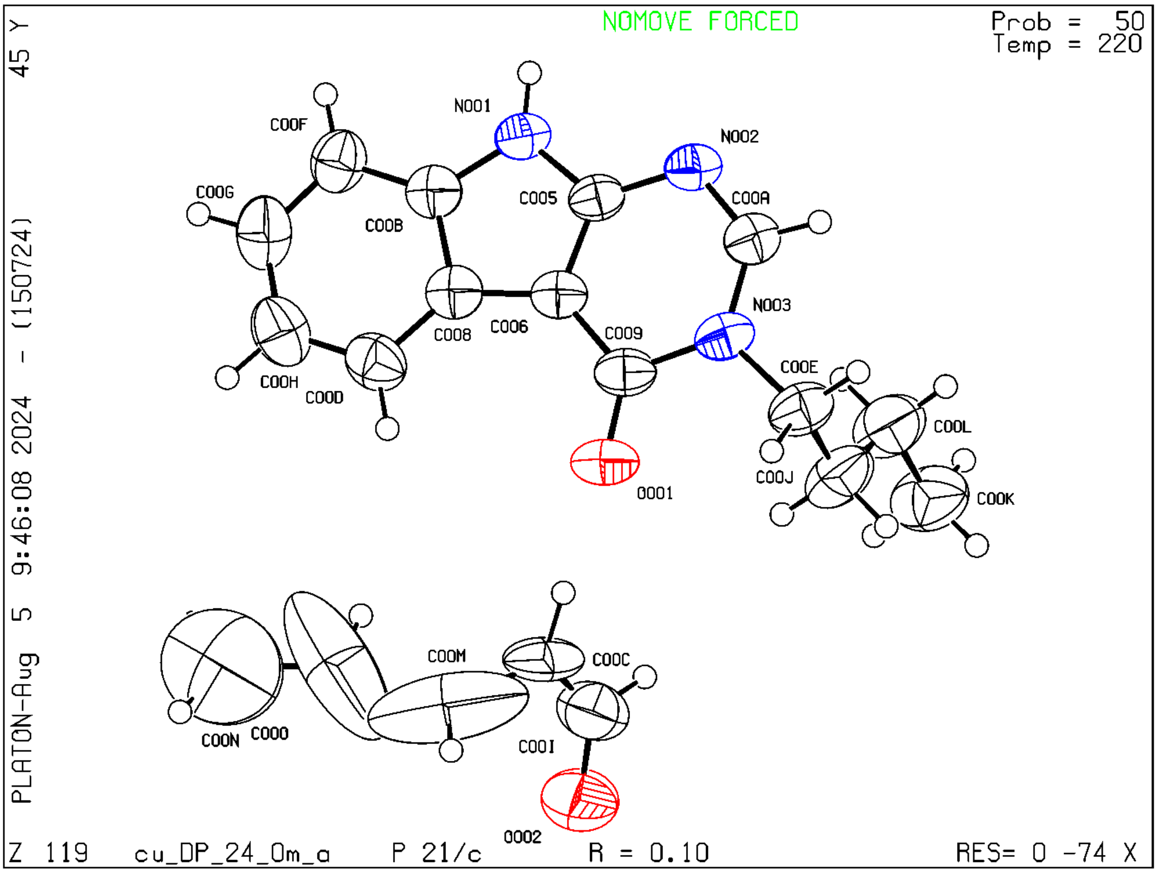

Supplement: File 2 — CheckCIF report for 7b. [file Beilstein_J_Org_Chem-21-217-s002.pdf]
